# Supplementary material for: Propagation and preservation of AI-discovered problem-solving strategies in human culture
Source: Nat Commun. 2026 Aug 18;17:8255. doi: 10.1038/s41467-026-76113-2 (PMC13487184; doi:10.1038/s41467-026-76113-2)
Supplement: Supplementary file 1 [file 41467_2026_76113_MOESM1_ESM.pdf]

Supplementary Information for

Propagation and preservation of AI-discovered problem-solving strategies in human culture

Levin Brinkmann[*†], Thomas F. Eisenmann[*†], Anne-Marie Nussberger[†],

Maxime Derex, Sara Bonati, Valerii Chirkov, Iyad Rahwan[*]

[*]Corresponding authors. Email: brinkmann@mpib-berlin.mpg.de (L.B.); eisenmann@mpib-berlin.mpg.de (T.F.E.); rahwan@mpib-berlin.mpg.de (I.R.)

[†]These authors contributed equally to this work.

Supplementary Text

Supplementary Note 1: Estimating Human Exploration Bias

To estimate bias in human exploration of the networks, we constructed a matched random counterfactual for each human trajectory. We focused on two discrete outcomes: the number of losses incurred along a solution path and the maximum level reached in the network. For each category k of an outcome variable x, we quantified bias using a one-vs-rest log-odds ratio comparing the probability that a human trajectory exhibits $x = k$ with the corresponding probability under the random counterfactual:

$$\mathrm{LOR}(k) = \log\left(\frac{P(x = k \mid \text{human})}{1 - P(x = k \mid \text{human})}\right) - \log\left(\frac{P(x = k \mid \text{random})}{1 - P(x = k \mid \text{random})}\right). \tag{S1}$$

Thus, $\mathrm{LOR}(k) = 0$ indicates no deviation from the random baseline, whereas positive and negative values indicate over- and under-representation of category k, respectively.

Uncertainty was assessed via nonparametric bootstrap resampling clustered at the participant level, resampling all trajectories from the same participant together. To avoid infinite log-odds in sparse categories, probabilities in each bootstrap replicate were estimated using Jeffreys smoothing, $\tilde{p}_k = (c_k + 1/2)/(n + 1)$, where c_k is the number of trajectories with $x = k$ out of n in the corresponding condition. Confidence intervals were obtained from the percentile range of the bootstrap distribution of $\mathrm{LOR}(k)$.

As shown in Fig. S1a, the distribution of losses is highly asymmetric relative to the random baseline. Zero-loss solutions are over-represented ($\mathrm{LOR}(0) = 4.39$, 95% CI $[4.13, 4.71]$; 1785 human vs. 51 random), corresponding to an increase in odds of more than two orders of magnitude. In contrast, trajectories involving multiple losses are systematically under-represented, with three losses ($\mathrm{LOR}(3) = -1.22$, 95% CI $[-1.38, -1.06]$; 260 human vs. 736 random), showing an approximately threefold reduction in odds.

Consistent with this pattern, Fig. S1b shows that human trajectories are biased toward shallow solutions. Level 0 is over-represented ($\mathrm{LOR}(0) = 3.33$, 95% CI $[3.16, 3.53]$; 1785 human vs. 144 random), whereas higher levels are progressively under-represented, most notably level 3 ($\mathrm{LOR}(3) = -1.86$, 95% CI $[-2.03, -1.70]$; 192 human vs. 924 random). Together, these results indicate that aversion to incurring losses constrains exploration to regions of the network with lower

level.

As shown in Fig. S2a, the qualitative pattern of loss aversion is stable across generations: zero-loss solutions are consistently over-represented, whereas trajectories involving multiple losses are under-represented. Participants in generation 0 had six exploration trials, whereas participants in later generations had only two. We find no statistically significant evidence that the additional trials attenuated this bias.

Within generation 0 (Fig. S2b), the bias against incurring losses increases over successive trials. Zero-loss solutions become progressively more over-represented, while trajectories with several losses are increasingly suppressed, indicating growing loss aversion even in the absence of inter-generational learning.

Supplementary Note 2: Classification of Populations

Figure S11 displays the average points of all participant in each population and generation. In the reward network task, a point value above 2000 is only possible if the largest level 3 is reached and multiple largest rewards of 400 points are collected. Therefore, we classify a generation of a population as 'discovered' if the average point value of the top performer exceeds 2000 points. Focusing on the last generation of each population, we found 9 groups exceeding this threshold in the human-machine population and one group in the human population.

Correspondingly, we found:

- **Permanently preserved:** In 9 out of 15 human-machine populations, humans permanently preserve the machine discovery.
- **Temporarily preserved:** In 6 out of 15 human-machine populations, humans temporarily preserve the machine discovery. This means that the machine discovery was preserved at least in the second generation; however, no participant in the last generation exceeded the threshold.
- **Discovered:** In 1 out of 15 human populations, the optimal strategy was discovered, and the maximum average point value in the last generation exceeds 2,000 points.

- **Not discovered:** In 14 out of 15 human populations, no participant exceeded an average point value of 2,000.

Supplementary Note 3: Qualitative Summary of Prototypical Strategies

Many written strategies referred to "looping" as a feature of the task that was related to higher performance, but not necessarily optimal: 'the trick is to identify a path between two nodes that both award high points and then repeat the path back and forth.', 'Find two letters with connecting green arrows and go back and forth between them.' Some participants admitted that they did not have a good plan of what to do: 'no idea seriously just choosing patterns', 'Unsure of an effective strategy'. Others confidently reported "superstitions" that focused on parts of the task that had no actual relation to good performance: 'remember the first random letters then remember a sequence', 'Longer routes seemed to provide more options for success'.

Frequently, written strategies explicitly referred to myopic behavior: 'Ignoring the big loss moves', 'Always avoiding neutral or negative paths seems to be the best strategy.' Sometimes, these myopic strategies even explicitly highlighted participants' bias against early losses: 'always choose the line that gives you points instead of taking away points for less risk', 'I just followed the green paths, I don't even really know what else I would try to do? Picking the negative point options makes no sense, after all.' In contrast, participants that had learned the loss strategy frequently referred to it explicitly and showed their long-term planning: 'Take a loss for the first three moves then move between two high scoring nodes.' 'if you take a hit early (eg the first 3 nodes are -50), you are in a position to maximize the following 7 nodes, each with +400. i tried it differently initially, but player 2's method is best.' The last example also emphasizes the important role of social learning for developing this participant's strategy.

Last, the one exceptional human player in the human populations that discovered the loss strategy on their own wrote 'I try o track my best point' (sic) after training, maybe indicating an approach that was open to exploring while systematically assessing their point score. After social learning, they wrote 'I tried to keep losing till I see the thinker green line.' (sic), clearly indicating the loss strategy even though no previous participant was able to demonstrate it to them. Presumably, the participant made good use of the "try yourself" trials to explore also unusual options, and discovered

the loss strategy this way, as the only player to do so in the 15 human populations.

One curious observation is that 7 out of 120 participants in generation 0 in human populations got coded as "mentioned the loss strategy" in the data. Writing down and coding written strategies is an inherently interpretative process, and it is unclear whether these participants just voiced suspicions, actually figured out the strategy, or whether this is just noise in the data or in the coding process. A qualitative inspection of the strategies suggests that they reasonably reflect the loss strategy, at least as defined by our coding manual. Most participants remained vague, e.g. "you have to reduce some points to gain some", suggesting that at least the exact implementation of taking three losses might have been hidden from them. Most importantly, the hunch that they might have had did not translate into better performance. The 9 players range from 1000 to 1600 points total, whereas we expected more than 2000 points for a player implementing the loss strategy (see "Humans Preserve the Machine Discovery"). Crucially, also their demonstration trials do not look much better, with only two select trials just scratching the 2000 point mark, but many failed attempts demonstrating 1500 points or less. So even by the end of the experiment, they were not able to demonstrate that they learned a superior strategy.

Supplementary Note 4: Task Performance (robustness checks)

Figure S11 shows the average reward per participants, separated by generation and population. Descriptively, human participants in the average human population started out equal to the average human-machine population in generation 0, but human-machine populations earned consistently higher rewards in generations 1 to 4.

We ran the linear mixed-effects model testing prediction 1a ("General Benefit of AI") on the demonstration trials of generations 1 to 4, with *total reward* as the dependent variable and fixed effects for *condition*, *generation*, and their interaction. Additionally, we included random intercepts for *participant* and *population*, as well as a random slope for *generation* on *population*. As preregistered, we centered and scaled *generation* to improve model convergence and make the main effect for *condition* interpretable. In line with our prediction, the model revealed a significant main effect for *condition* ($\beta = -309.2$, $SE = 57.0$, $CI_{95\%} = [-418.4, -200.1]$), indicating that human-machine populations outperformed human populations overall.

The model for prediction 1b (”Long-lasting Benefit of AI”) followed a similar logic, but was using only the data of generation 4. Given the more restricted data set, only the fixed effect for *condition* and the random intercepts for *participant* and *population* remained. Similar to the first model, there was a meaningful main effect for *condition* ($\beta = -229.1$, $SE = 82.4$, $CI_{95\%} = [-383.1, -55.5]$), supporting our prediction that human-machine populations would perform better than human populations by the last generation.

The model for prediction 2a (”Transmission from AI to Humans”) was identical to the one for 1b, but used the data of generation 1. Again, there was a significant main effect for *condition* ($\beta = -404.6$, $SE = 59.4$, $CI_{95\%} = [-524.8, -286.1]$), supporting our prediction that human-machine populations performed better than human populations in the immediate generation learning from machine agents. Here, the model returned a warning about a singular fit, so we also checked all other optimizers available in `lme4`; however, since all optimizers converged to identical values for the fixed effects, we concluded the warning to be false-positive.

Human-Machine Behavioural Congruency

Figure S11.B shows the proportion of human moves congruent with machine-generated moves per demonstration trial, summarized for each participant. Qualitatively, the results are similar to the results for rewards: no statistically significant difference between conditions in generation 0, but there is consistently more behavioural congruency in human-machine populations for generations 1 to 4.

The models assessing predictions 1a to 2a were identical to the ones for reward, with exception of the different outcome measure and being on the level of moves per trial. Since behavioural congruency was a binary variable, we used a logit link here. All three models replicated the expected effects for condition found for reward on the behavioural congruency measure: Human-machine populations were behaviourally more congruent than human populations overall ($\beta = -0.87$, $SE = 0.14$, $CI_{95\%} = [-1.12, -0.61]$), in the last generation ($\beta = -0.64$, $SE = 0.20$, $CI_{95\%} = [-1.02, -0.25]$), and in the first generation ($\beta = -1.13$, $SE = 0.13$, $CI_{95\%} = [-1.41, -0.88]$). Again, the last model returned a warning about a singular fit, but all available optimizers converged to identical fixed effects until the second digit, so we took it as a false positive.

Written Strategies

Figure 4.C shows the proportion of human participants reporting the loss strategy, summarized by generation and population. In the human populations, we observe practically no mention of the loss strategy, with proportions close to 0 throughout all 5 generations; the exception to this is the single population that discovered the loss strategy towards the end, resulting in 50% of the participants referring to the strategy in generation 4. In contrast, participants in the human-machine populations consistently mentioned the loss strategy explicitly, with proportions of about 30% to 50% per generation. In generations 1 to 4 (i.e. with social learning), almost no participants mentioned the loss strategy immediately after the training trials (human populations: 1.7%; human-machine populations: 2.7%), but 39.8% of participants in the human-machine populations mentioned it after social learning had occurred (compared to 1.9% in the human populations). In comparison, in generation 0 (without social learning) very few participants in either condition referred to the loss strategy at either time point (human-machine t1: 1.3%; t2: 1.3%; human-only t1: 2.5%; t2: 5.8%).

The model assessing prediction 2b ("Explicit Recognition of Counterintuitive Strategy") was identical to the one for prediction 1a, but using a logit link due to the binary outcome variable and missing the random intercept for participants, since it was built on participant level. Again, there was a significant main effect for condition ($\beta = -4.18$, $SE = 0.62$, $CI_{95\%} = [-6.30, -3.11]$), indicating that human-machine populations explicitly mentioned the loss strategy more frequently than human populations from generations 1 to 4. This model also returned a warning about a singular fit, but all available optimizers converged to qualitatively similar parameters.

Robustness Checks

As a robustness check, we revisited the random-effects structure in Models 1a and 2b. In the original specification, generation was treated as a continuous predictor and incorporated via a random slope, but not explicitly modeled as a grouping factor. We therefore re-estimated the models with generations nested within populations, replacing the fixed effect of generation. In Model 1a, participants were additionally nested within generations (this structure was not feasible in Model 2b). All models converged under this alternative specification, and the main effect of condition remained robust:

- **Performance Model 1a**: $\beta = -309.2$, $SE = 40.3$, $\text{CI}_{95\%} = [-418.0, -195.4]$
- **Congruency Model 1a**: $\beta = -0.87$, $SE = 0.14$, $\text{CI}_{95\%} = [-1.14, -0.60]$
- **Written Strategy Model 2b:** $\beta = -4.24$, $SE = 0.59$, $\text{CI}_{95\%} = [-5.91, -3.29]$

Supplementary Note 5: Agent-Based Model

We developed a parsimonious agent-based model capturing the essential components of our multi-generational cultural transmission experiment. Each generation implements the following sequential processes: individual learning, reward-based teacher selection, social transmission, strategy integration, demonstration, and reward updating.

Each simulation consists of $M = 50$ independent replications, $G = 20$ generations, and $N_{\text{gen}} = 20$ agents per generation. Depending on the experimental condition, a subset $N_{\text{m}} = 5$ of agents are labeled as machines, characterized by distinct exploration budgets K_{m} and optimality biases q_{m}, which are systematically manipulated.

Individual learning. Each agent labeled as human performs $k_{\text{h}} = 10$ learning trials, allocated between exploration of optimal and myopic strategies according to an exploration bias $q_{\text{h}} = 0.01$, chosen to investigate a regime with a considerable bias toward the myopic solution:

$$k_{\text{opt}} \sim \text{Binomial}(k, q_{\text{opt}}), \quad k_{\text{myo}} = k - k_{\text{opt}}. \tag{S2}$$

Let d_{opt} denote the per-trial discovery rate of the optimal strategy, which is experimentally manipulated, and let $d_{\text{myo}} = 0.5$ denote the discovery rate of the myopic strategy. The cumulative discovery probabilities are given by:

$$D_{\text{opt}} = 1 - (1 - d_{\text{opt}})^{k_{\text{opt}}}, \quad D_{\text{myo}} = 1 - (1 - d_{\text{myo}})^{k_{\text{myo}}}. \tag{S3}$$

Agents stochastically discover either an optimal solution, a myopic solution, or no solution. If both solutions are discovered, priority is given to the optimal solution.

Demonstration and rewards. Agents produce K_{demo} independent demonstrations. Let n_{opt} and n_{myo} be the numbers of optimal and myopic demonstrations (the remainder are null). Each demonstration is perturbed by independent noise $\epsilon_k \sim \mathcal{N}(0, \epsilon^2)$. The total reward is

$$R = n_{\text{opt}} R_{\text{opt}} + n_{\text{myo}} R_{\text{myo}} + \epsilon_{\text{eff}}, \quad \epsilon_{\text{eff}} \sim \mathcal{N}\left(0, \epsilon^2 K_{\text{demo}}\right), \tag{S4}$$

where $R_{\text{opt}} = 1$, $R_{\text{myo}} = 0.5$, and $\epsilon = 0.1$.

Teacher selection and social learning. For generations $g > 0$, each learner samples k candidate teachers uniformly at random from the previous generation. Teachers are selected according to a softmax function over their observed rewards:

$$p_i \propto \exp(r_i/\tau), \tag{S5}$$

where τ controls the intensity of selection.

Learners adopt the selected teacher's optimality state with probability λ. Individual and social learning outcomes are integrated according to:

$$s_g = \max(s_{\text{ind}}, s_{\text{social}}), \tag{S6}$$

ensuring that optimal strategies dominate myopic or null strategies whenever they are acquired. In the initial generation ($g = 0$), learning proceeds exclusively through individual exploration.

Simulated conditions. We first investigated the ability of homogeneous populations to discover and maintain the optimal strategy as a function of exploration capacity (k_{h}), bias toward myopic exploration (q_{h}), optimal solution discoverability ($-\log_{10} d_{\text{opt}}$), and social learnability ($1 - \lambda$). In Figure S12, we report the probability of discovery across populations persisting for 1, 5, and 20 generations.

We then focused on scenarios with heterogeneous exploration capacity and bias, in which the majority of agents (i.e., human agents) are limited in their exploration capacity ($k_{\text{m}} = 10$) and exhibit a bias ($q_{\text{m}} = 0.01$) toward myopic solutions. We introduced five machine agents in the first generation and systematically manipulated their exploration capacities ($k_{\text{m}} \in 10, 1000, 100000$) and biases toward optimal strategies ($q_{\text{m}} \in 0.01, 0.5$). Figure 5 shows the fraction of agents adopting

the optimal strategy in the final generation as a function of social learnability $(1 - \lambda)$ and optimal solution discoverability $(-\log_{10} d_{\text{opt}})$.

Next, we varied the strength of social learning selection $\tau \in [0.1, 0.2, 0.4]$ and examined different machine-placement scenarios (no machines, machines only in the first generation, and machines in all generations; see Figure S13). Similarly, we varied the noise level applied to individual realized rewards $\epsilon \in [0.1, 0.2, 0.4]$ (see Figure S14).

Finally, we assessed the robustness of the main findings for high-capacity $k_{\text{m}} = 100000$, unbiased $q_{\text{m}} = 0.5$ machines with respect to the number of machines in the first generation $N_{\text{m}} \in [1, 5]$ and the total population size per generation $N_{\text{total}} \in [20, 100]$ (see Figure S15).

Supplementary Note 6: Pilot Studies

We run 10 cycles of pilots. Each in each cycle we run some of a set four different types of pilots indicated by the letters A to D. These type of pilots differ in the experimental setup and therefore allowed to test different premises.

- **Pilot type A**: Participants are placed in generation 0 of the experiment. They therefore have no chance of social learning. This pilot allows conclusion about the discoverablity of the loss strategy.

- **Pilot type B**: Participants are placed in the first generation of the experiment. Solutions of the first generation are provided by rule based algorithm. Generally, we provided participants with a mix of teachers. Some of the teacher would show a solution of the loss strategy while other teacher would display a myopic solution. This pilots allows to infer about the learnability of the solution, provided the participant is exposed to the loss strategy. This type of pilots also allows to infer about the selection of a teacher by the participant.

- **Pilot type C**: This pilot type was similar to pilot type B, however, participant could only choose from myopic solutions to learn from.

- **Pilot type D**: This pilot type was the most complex of all. Here we collected data from the 0th and 1st generation. No machine solution was provided. Like pilot A, this pilot was

intended to investigate the learnability. Additionally this pilot allowed a technical check of the multi-generational assignment.

Not all pilots where informative. In the following we present a selection of all pilots.

- **Pilot 1 A / B**
  - **Setup**: The network task presented in these earlier pilots is similar to the final reward network task, with important differences:
    - The full network is shown to participants, but humans are expected to behave suboptimally due to cognitive constraints in exploring the full solution space.
    - The network is smaller, with only 8 nodes.
  - **Results**: Participants fail to discover the loss strategy. Even when shown the loss strategy, they fail to repeat it.
- **Pilot 2 A / B**
  - **Setup**:
    - Increased the time for participants.
    - Made it easier for participants to reproduce the correct strategy by showing it in written form.
    - Asked participants to write down their strategies.
  - **Results**:
    - In Pilot 2 A, participants enacted and described a myopic strategy.
    - In Pilot 2 B, only 4 out of 10 participants were able to reproduce the loss strategy, either in practice or verbally, even after explicit instruction.
- **Pilot 3 B**
  - **Setup**: Focused on the learnability of the loss strategy:
    - Intensified the social learning phase.

    - * Introduced iterative observation and try-yourself pages, allowing participants to play the task themselves and compare their performance to the teacher's.
  - **Results**: Only 2 out of 10 participants somewhat learned the loss strategy.

- **Pilot 4 B**
  - **Setup**: Made the written correct strategy even more salient.
  - **Results**: No significant improvement in learnability.

- **Pilot 5 B**
  - **Setup**:
    - * Simplified the task.
    - * Redesigned the networks to better separate the myopic and loss strategies in terms of point values.
  - **Results**: Most participants learned the loss strategy and were able to reproduce it in demonstration trials.

- **Pilot 5 A / C**
  - **Setup**:
    - * Conducted two more pilots with the same experimental design as Pilot 5 B.
    - * In Pilot 5 C, participants could socially learn but only had myopic solutions to choose from.
    - * In Pilot 5 A, participants learned only individually.
  - **Results**: Performance in both pilots was mixed. While fewer participants used the loss strategy, some did. This was deemed a failure, as the goal was for no (or very few) participants to discover the loss strategy on their own.

- **Pilot 6 B**
  - **Setup**:
    - * Redesigned the task and experimental flow to prevent backward induction.

    * Made unexplored parts of the networks hidden, reflecting real-world constraints like those in Go.
    * Enhanced the social learning phase to highlight the exploration advantage of the machine.
  - **Results**: Promising results, with 3 out of 10 participants understanding the loss strategy but struggling to execute it.

- **Pilot 7 B**
  - **Setup**: Improved the UI in the social learning phase to:
    * Make potential strategies more salient when the teacher shows the solution.
    * Emphasize the contrast between participants' suboptimal solutions and the teacher's optimal solution.
  - **Results**: Five out of 10 participants somewhat learned the strategy, but many struggled to reproduce it.

- **Pilot 8 A / B**
  - **Setup**: Redesigned the network task to:
    * Remove links from higher levels back to the first level, which tempted participants to avoid losses.
    * Ensure losses only occurred when moving up levels, with the largest gains exclusive to the highest level.
  - **Results**:
    * Pilot 8 B: Six out of 10 participants successfully implemented the loss strategy, with three doing so without error.
    * Pilot 8 A: Confirmed that participants could not discover the loss strategy on their own.

- **Pilot 9 D**

  - **Setup**: Tested the task with more participants (N = 51) to ensure the strategy was not discoverable.
  - **Results**: None of the participants discovered the loss strategy.

- **Pilot 9 B**
  - **Setup**: Ran another 10 participants in the B configuration.
  - **Results**: Only 7 out of 20 participants learned the strategy. Some networks where the loss strategy was not superior to the myopic strategy confused participants.

- **Pilot 10 B**
  - **Setup**: Removed networks where the loss strategy performed worse than the myopic strategy.
  - **Results**: Nine out of 20 participants imitated the loss strategy without error.

Through this piloting, we calibrated the task such that the optimal strategy's complexity was not trivial but also not impossible for human minds to discover and feasible to learn. For instance, in an early set of pilot studies, a few participants were able to discover the optimal strategy through backwards induction. To more realistically reflect that backwards induction is prohibitively costly and/or impossible due to the sequential ordering of effects in real-world problems, our final task consisted of networks in which paths were revealed sequentially according to players' moves. Illustrating the defining influence of learnability, in pilots where the optimal strategy involved incurring a precise number of large losses (e.g., no more or less than three), participants struggled to learn this within the limited time available in our experiment. Our final task thus involved a simplified optimal strategy of taking losses whenever presented with one.

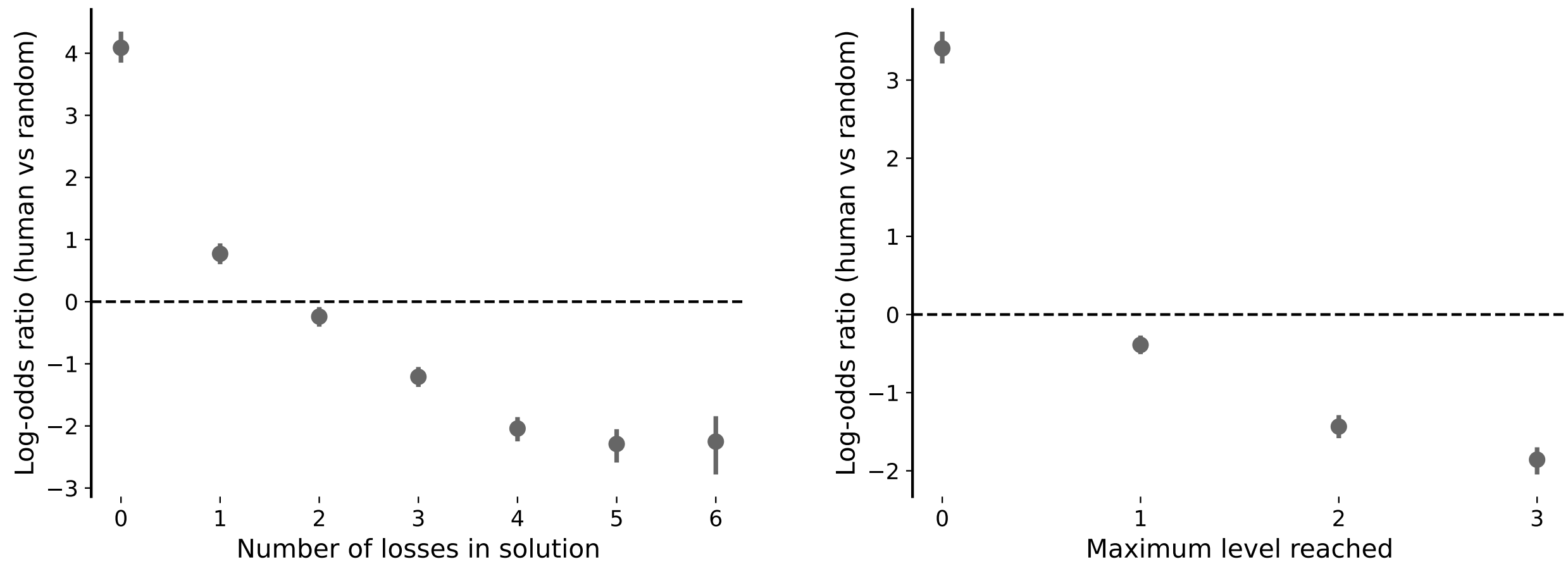

***(a)** Number of losses.*

***(b)** Maximum level reached.*

***Figure S1: Loss Aversion in Human Exploration.** Log-odds ratios comparing human and random-counterfactual distributions for (a) the number of losses incurred and (b) the maximum level reached, based on N = 1,155 participants. Points show $\mathrm{LOR}(k)$ and error bars indicate 95% bootstrap confidence intervals (CI; clustered by participant and using Jeffreys smoothing). The dashed line at zero indicates no bias (i.e., the human distribution matches the random counterfactual).*

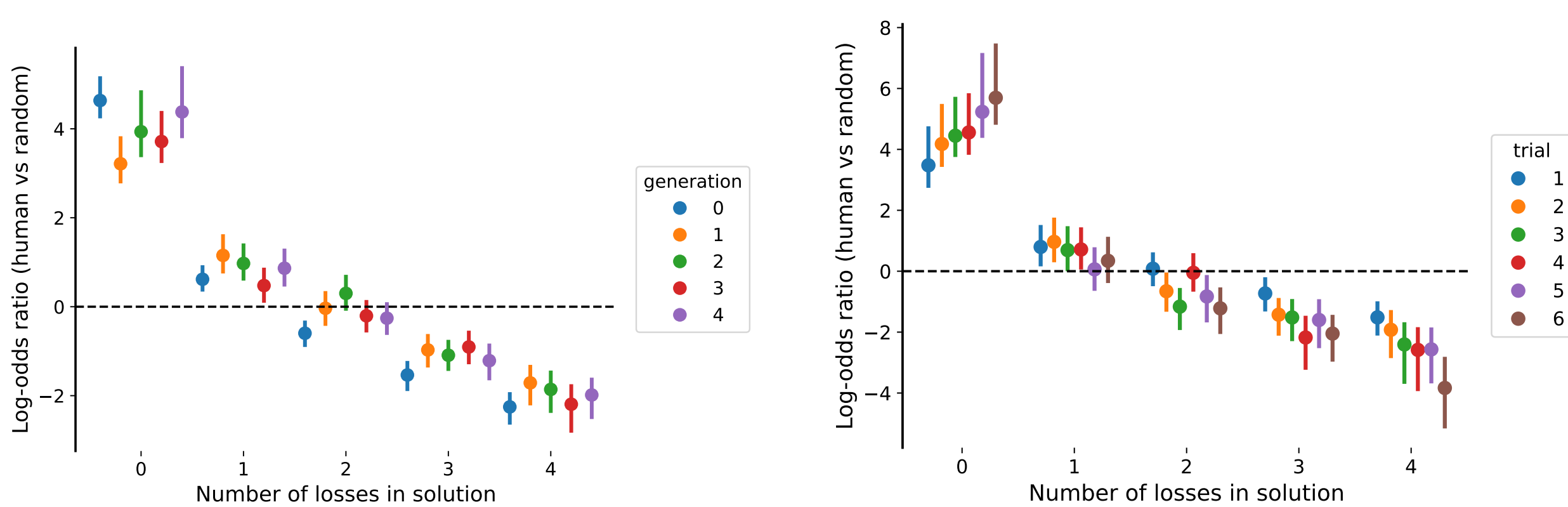

***(a)** By generation.*

***(b)** Across trials in generation 0.*

***Figure S2: Loss Aversion Across Generations and Trials.** Log-odds ratios for the number of losses, shown (a) separately for each generation (n = 195 human participants in generation 0 and n = 240 in each of generations 1–4) and (b) for each of the six learning trials in generation 0. Error bars indicate 95% bootstrap CI (clustered by participant). The dashed line at zero indicates no bias (i.e., the human distribution matches the random counterfactual).*

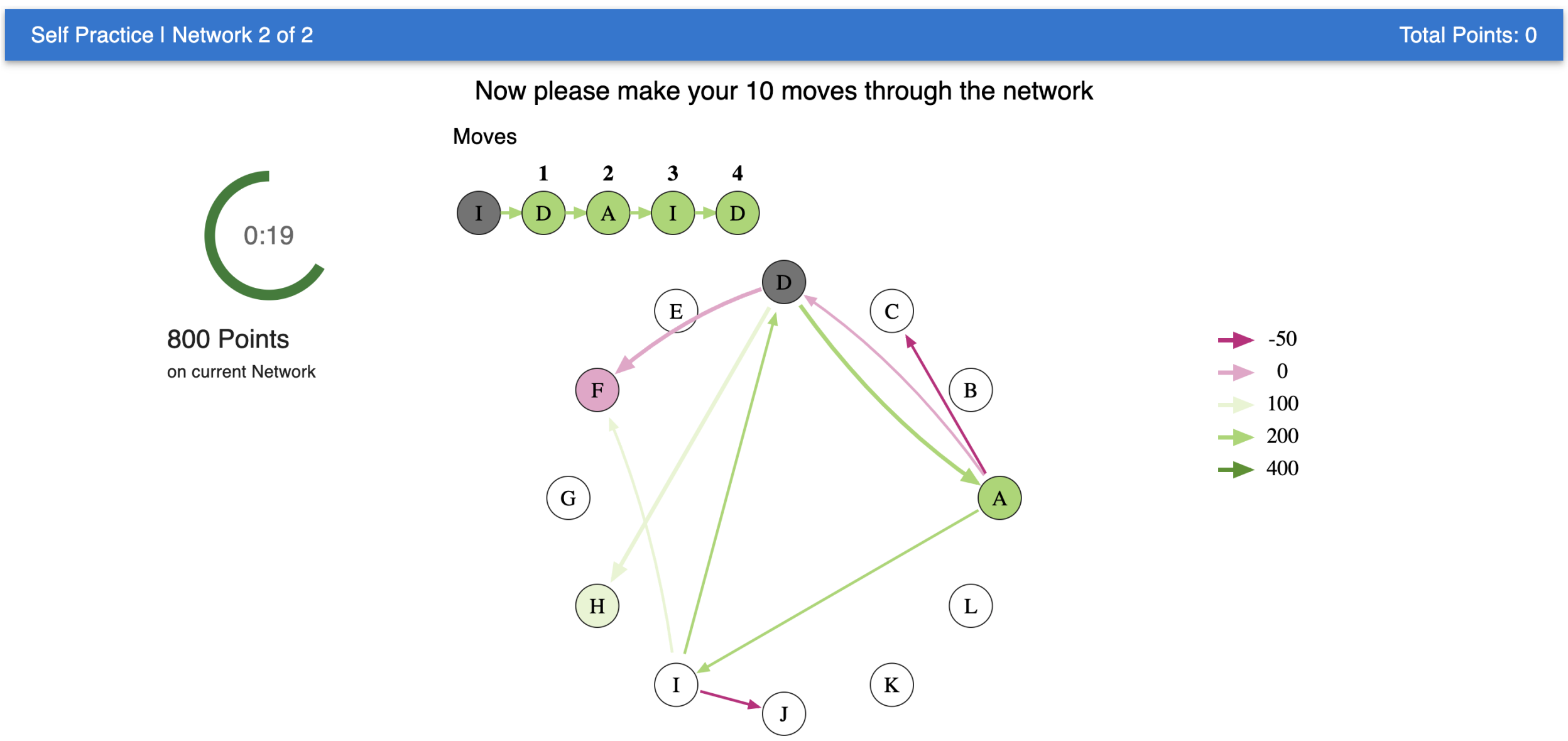

Figure S3: Self-learning page. *In the first part of the experiment, participants are on their own to solve the full version of the task. This phase is of different length depending on the generation. In generation 1-4, participant can try themselves on two different networks. The main purpose here is for participants to get an better understanding of the task, before entering the social learning phase. In generation 0, participants have no social learning phase. Correspondingly, for these participants the self-learning phase is extended to a total of 6 networks.*

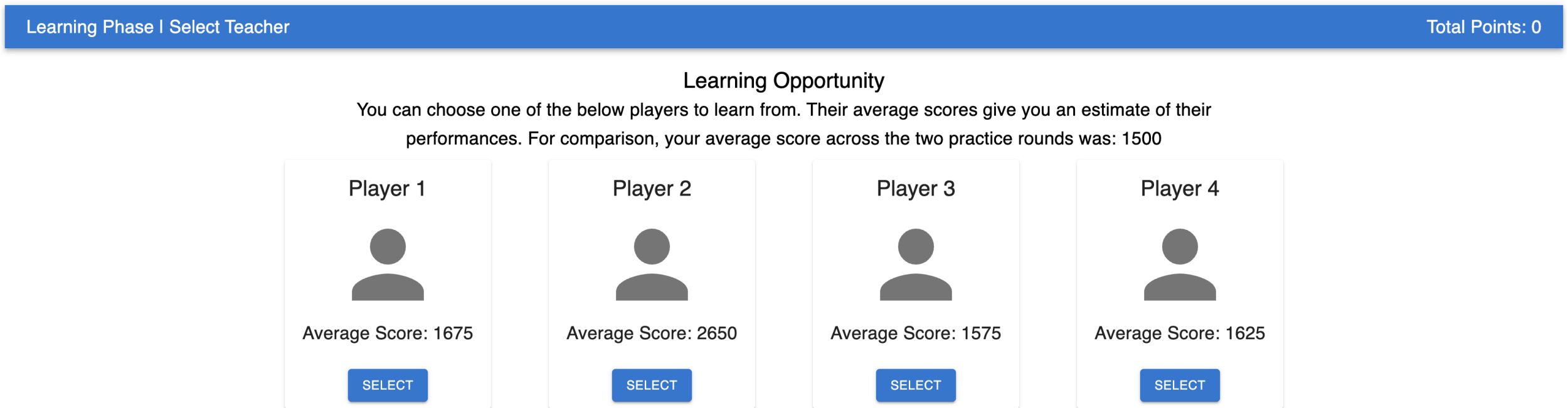

Figure S4: Select demonstrator. *Before entering the social learning phase, participants can select one out of five potential demonstrators. The only information guiding participants' selection is the demonstrators' average score, which is computed from the points collected during the demonstrators' demonstration phase. Otherwise, each demonstrator is symbolised identically, with no discrimination between human and machine player. Participants were informed that demonstrators could be humans or machines, however. As an additional comparison to make their choice, participants' own average performance on the previous two self-learning networks is displayed.*

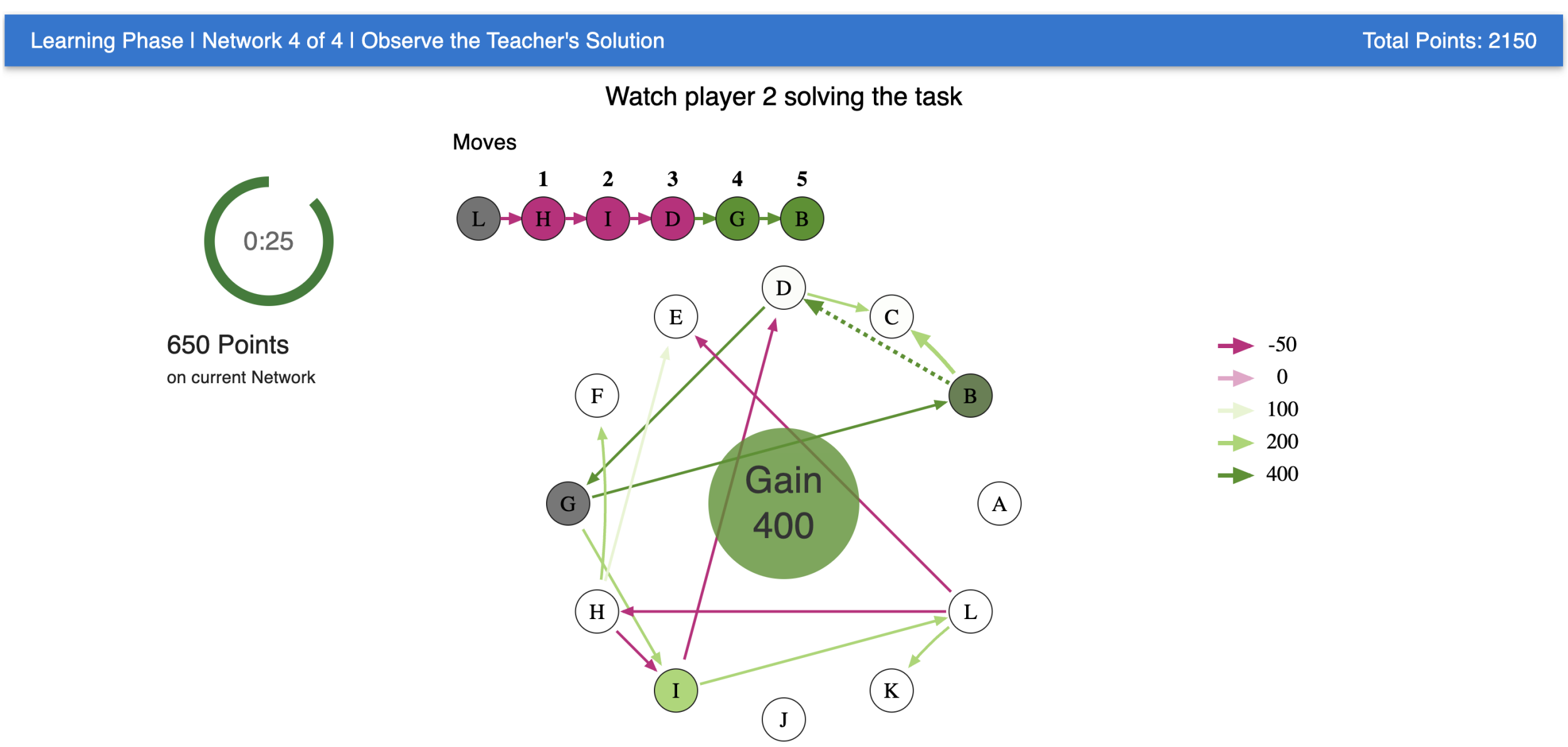

Figure S5: Social learning by observation. *Participants see a replay of the demonstrator solving the task. The task is displayed in the same way as during self-play, however the UI is deactivated. For each step of the solution, the next move is indicated as a dashed line.*

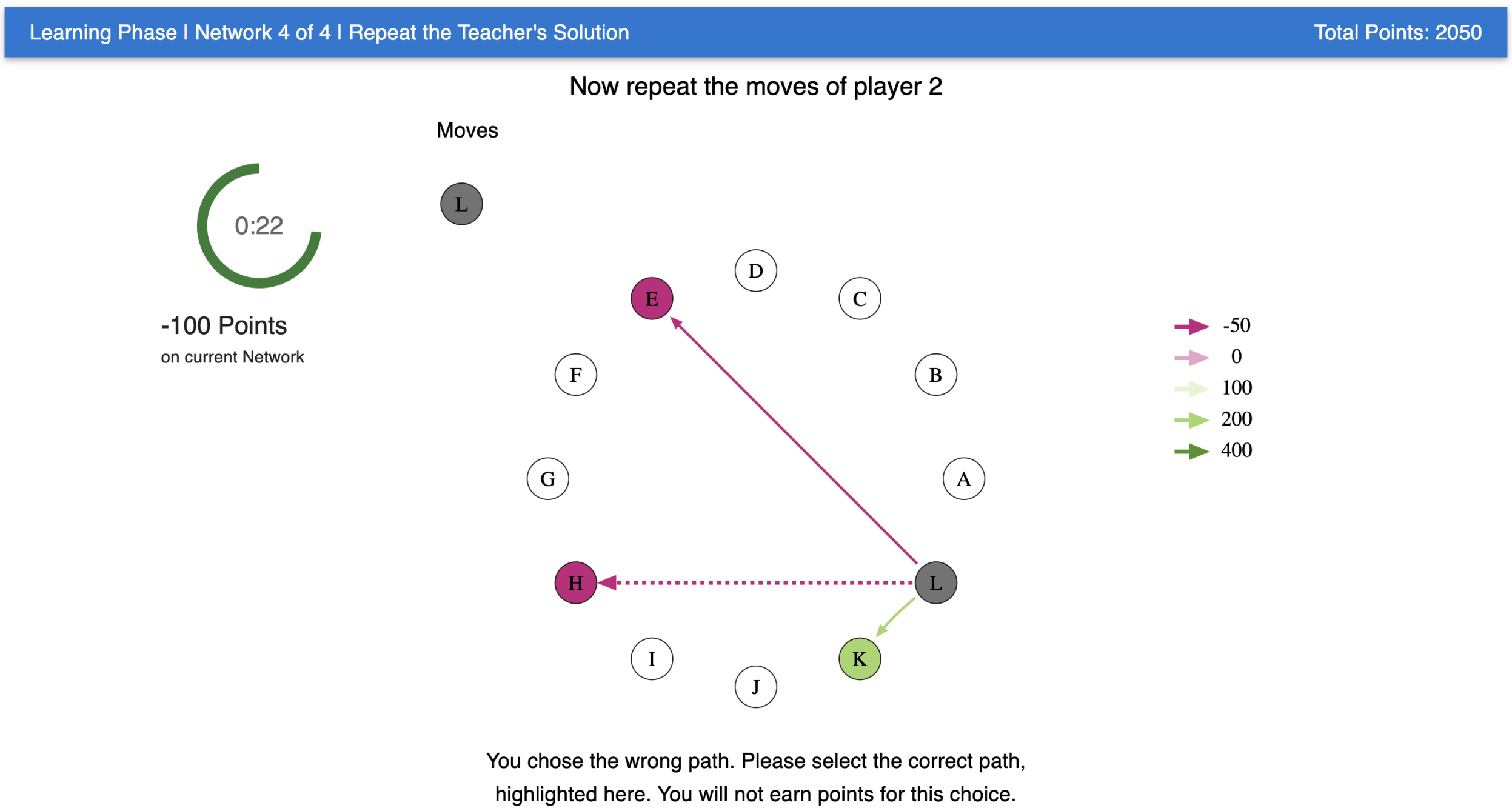

Figure S6: Social learning by repetition. *Participants are exposed to the same network and are asked to repeat the demonstrators' moves. Participant are receiving +100 points for correct repetitions and -100 points for wrong repetitions. If a wrong move is chosen, the right move is indicated by a dashed line. Participants still have to enact the right move, but do not receive points.*

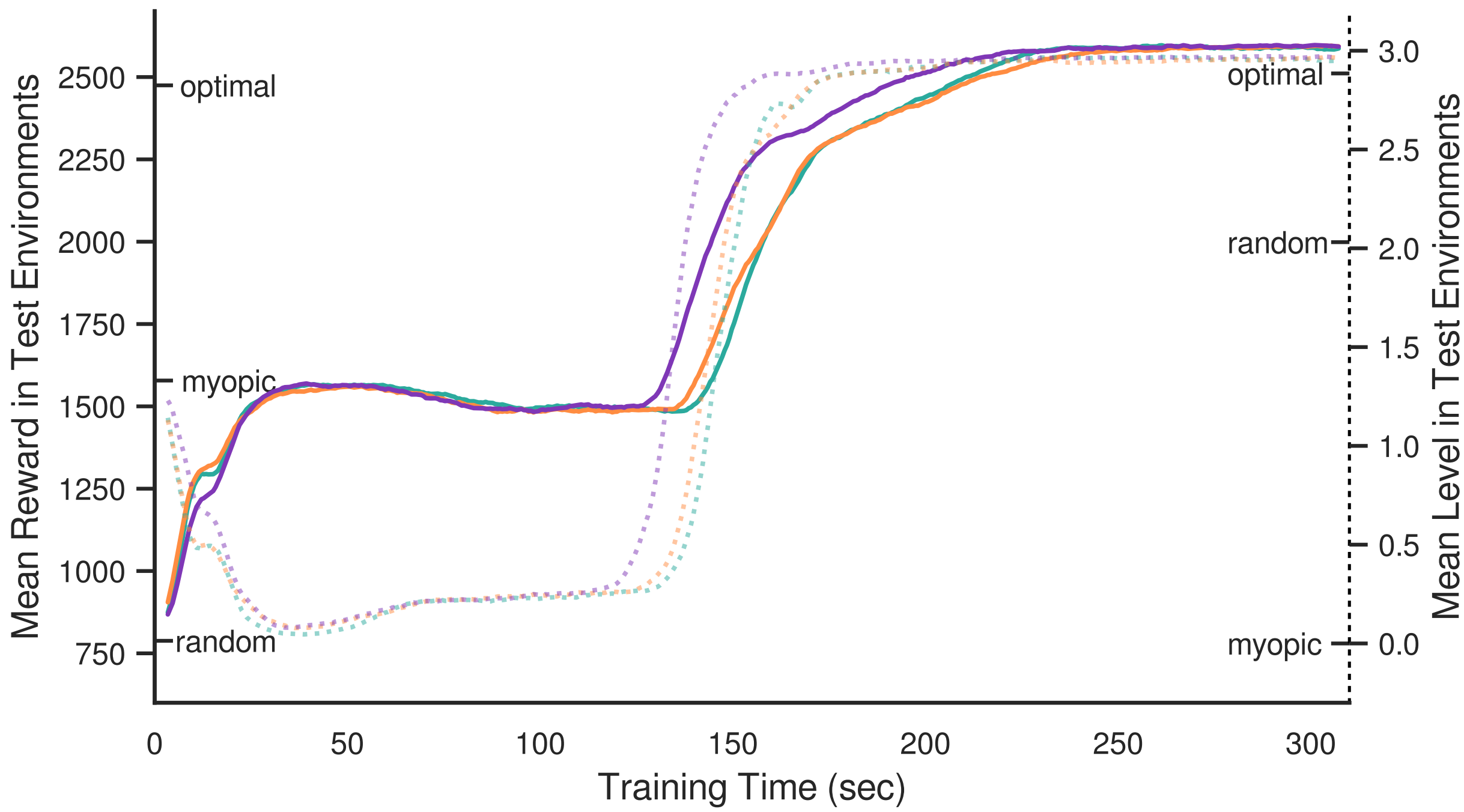

Figure S7: Algorithmic performance during training. *We trained three Deep Q-learning algorithms – signified by the three distinct colours – during a time period that was similar to the Individual Learning Phase for human participants (i.e., 315 seconds) for 5,000 episodes on 10,000 unique networks (distinct from the experimental ones). The three colors correspond to three independent training runs with different random seeds (seeds 0, 1, and 2). The left y-axis references the solid lines which denote the algorithmic agents' achieved rewards. The right y-axis and dashed lines denote the achieved maximum level, where the highest level could only be reached by incurring losses to progress. We marked the average values for three heuristic benchmarks on both y-axes: a random agent, a myopic agent always choosing the highest immediate reward, and a loss-seeking agent pursuing losses when possible. During training, the algorithms first discover the myopic strategy, before finally converging on the optimal strategy.*

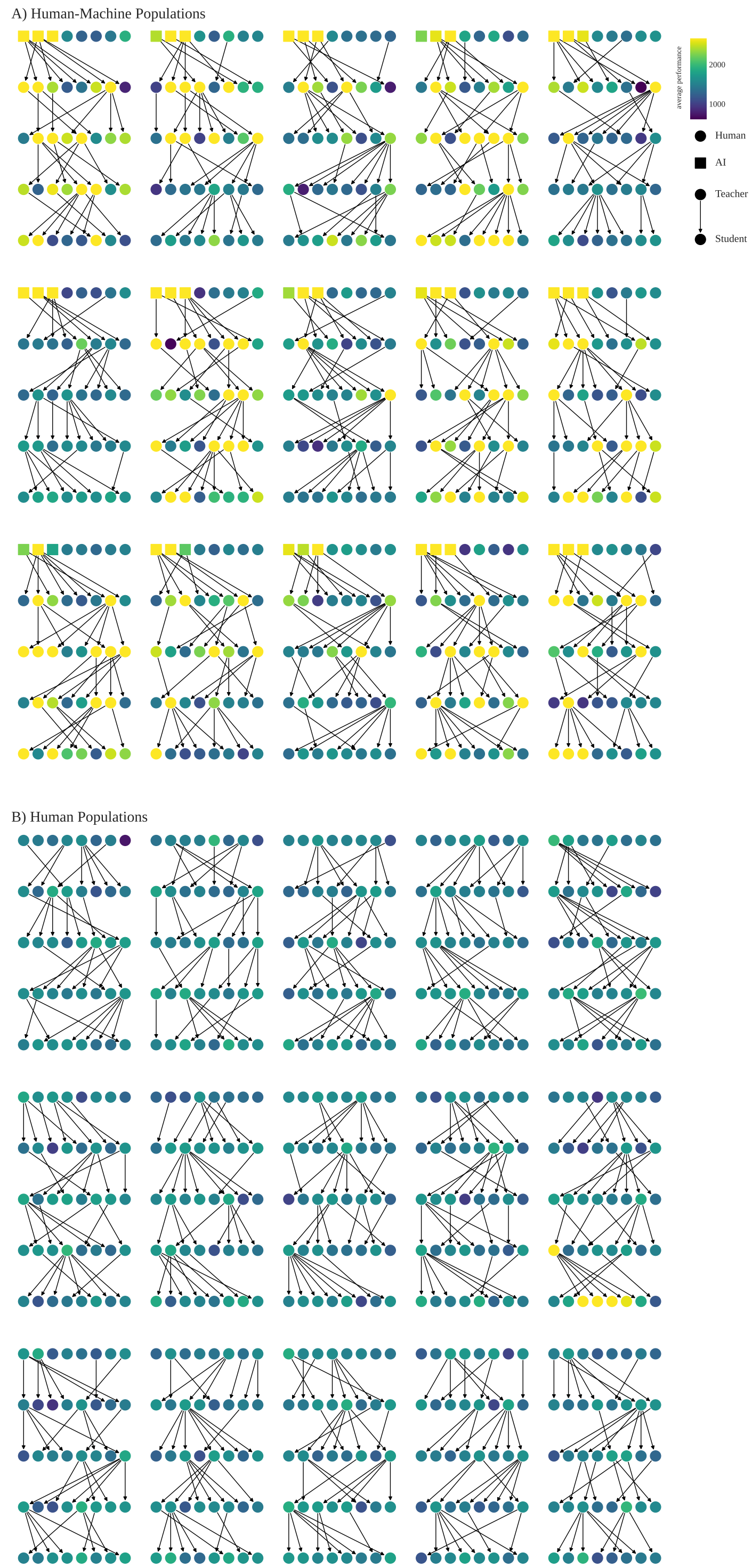

Figure S8: Performance of All 30 Populations*: Each population consists of five generations, each with eight players. In human-machine populations, there are three machine players in the first generation. Human players are represented by circles and machine players by squares, with the color of each symbol indicating the player's average score. The color scale spans the empirical range of average scores across all players, with lighter shades corresponding to lower scores and darker shades to higher scores. In generations following the first, each player selects a single teacher. Arrows depict these teacher-student relationships.*

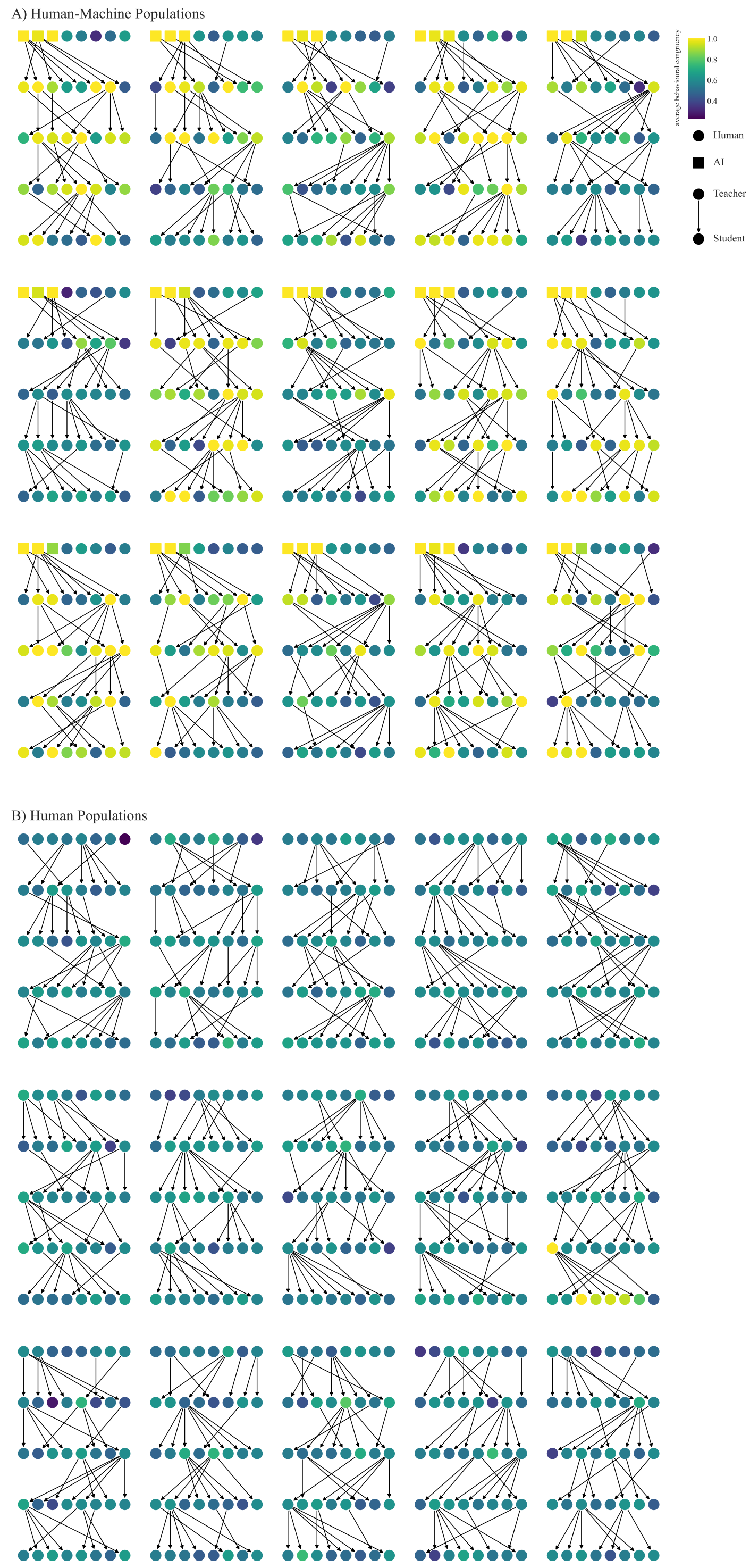

Figure S9: Behavioural Congruency of All 30 Populations*: Human players are represented by circles and machine players by squares, with the color of each symbol indicating the fraction of the player's moves that are congruent with those of the machine. The color scale ranges from 0 (light) to 1 (dark). A move is considered congruent if, given the same context (including historical actions), the machine would have made the same move. Arrows depict teacher-student relationships.*

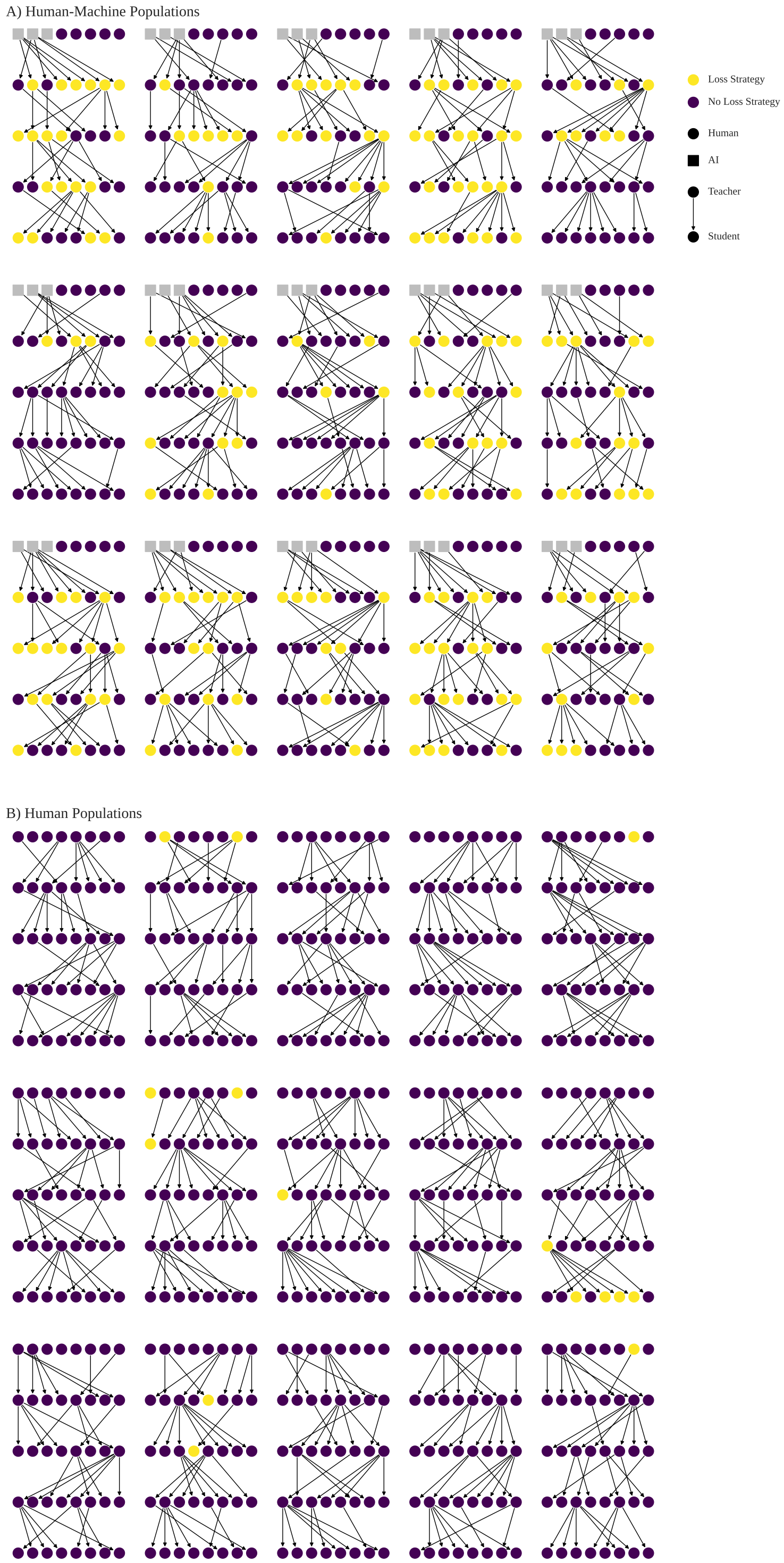

Figure S10: Coded Strategies of All 30 Populations*: Human players are represented by circles and machine players by squares. A yellow circle signifies that the human player, after social learning, described a written strategy resembling deliberate acceptance of losses. Other strategies are represented by dark violet. Arrows depict teacher-student relationships.*

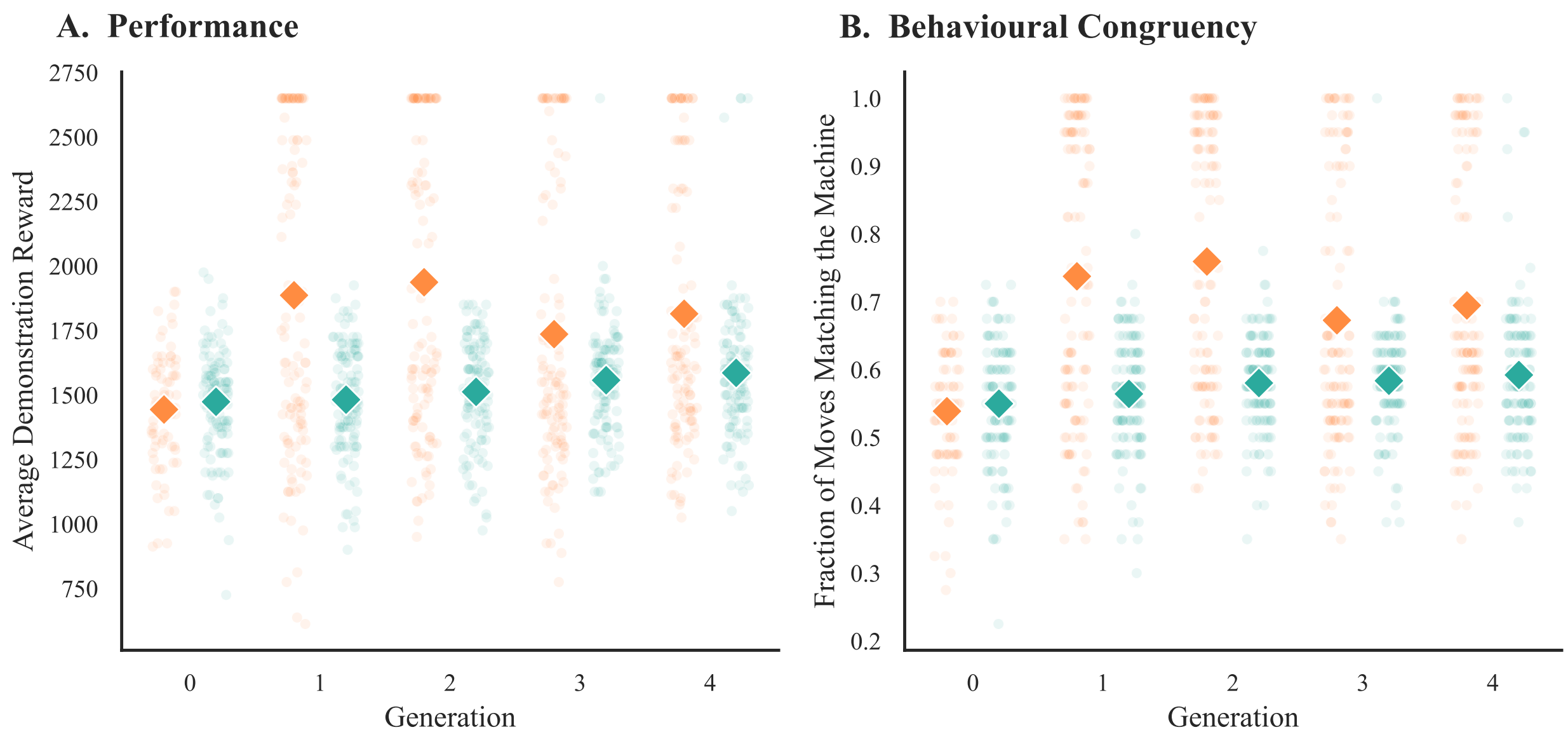

Figure S11: Individual Task Performance and Behavioural Congruency*: Individual participants are represented by dots, and the grand mean for each condition is indicated by a diamond. Human-only populations are shown in green (n =* 600 *human participants across 15 populations), and human–machine populations in orange (n =* 555 *human participants across 15 populations). Performance (Panel A) refers to the average number of points earned per participant during the Demonstration Phase. Behavioural congruency (Panel B) is defined as the proportion of moves made by a human player that would also have been made by a machine player, conditional on identical preceding moves. Machine behaviour is operationalised using the majority vote across three independently trained models.*

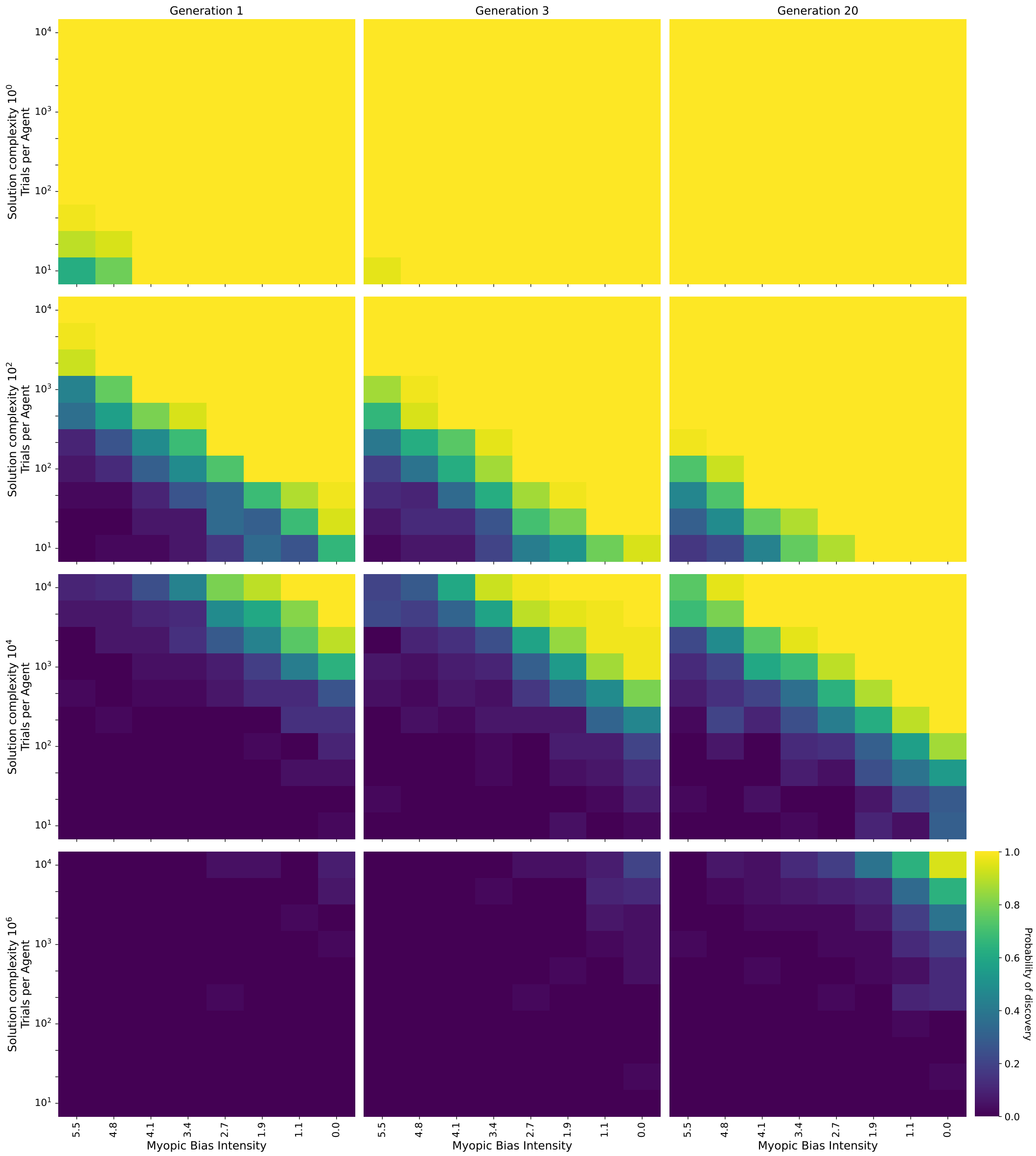

Figure S12: Discovery probability as a function of exploration capacity and bias. *We simulated 20 agents over 20 generations, with each agent assigned a fixed number of trials per generation, as indicated by the x-axis of each subplot, and systematically varied the exploration bias toward myopic strategies, as indicated by the y-axis. Panels in different rows represent increasing solution complexity, while columns indicate the number of generations elapsed. Social learning parameters were held constant ($\tau = 0.1$ and $\lambda = 0.9$). Brighter colors indicate a higher probability that populations have discovered the optimal strategy. The heatmaps suggest that exploration capacity K and bias q constrain the discoverable solution complexity, with intergenerational transmission partially mitigating these constraints.*

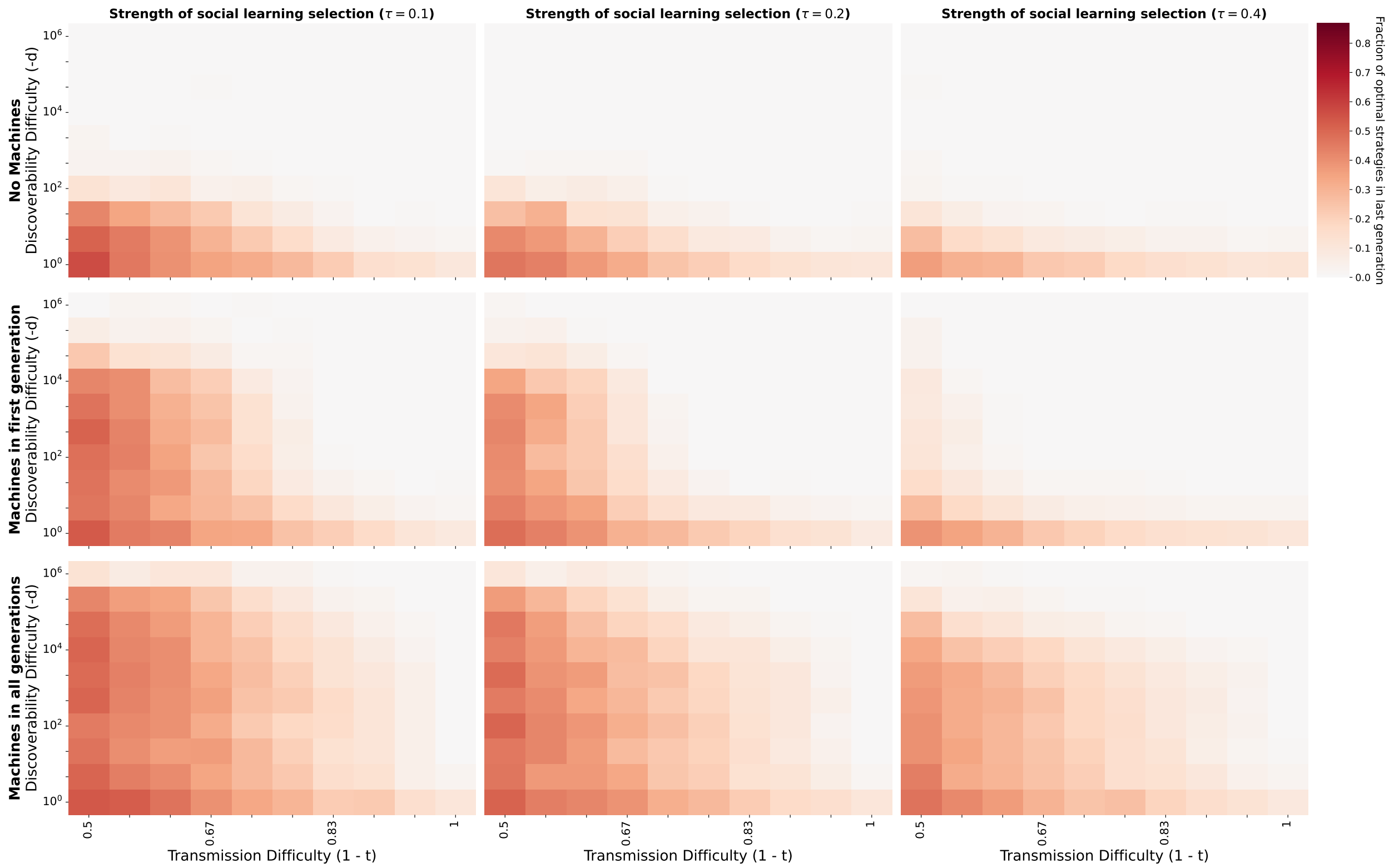

Figure S13: Selective social learning and machine availability. *We investigate how the strength of social learning and the availability of machines influence the adoption of the optimal solution. Social learning strength is operationalized via a softmax function over the performance of potential teachers, with lower temperature τ indicating stronger selection toward higher-performing teachers. We simulated 20 agents over 20 generations. We compare scenarios with no machines, five machines in the first generation only, and five machines present in all generations. Machine advantage and bias are set to* $K_{\text{machine}} = 10000$ *and* $q_{\text{machine}} = 0.5$*, respectively. Color indicates the fraction of human agents adopting the optimal strategy in the final generation, with the color scale ranging from 0 (light) to 1 (dark). Results suggest that stronger selective social learning enables populations to adopt strategies that are more difficult to discover and transmit. Selective social learning is particularly important for preserving high-performing, machine-discovered strategies across generations. By contrast, selective social learning is less consequential when machines are consistently present.*

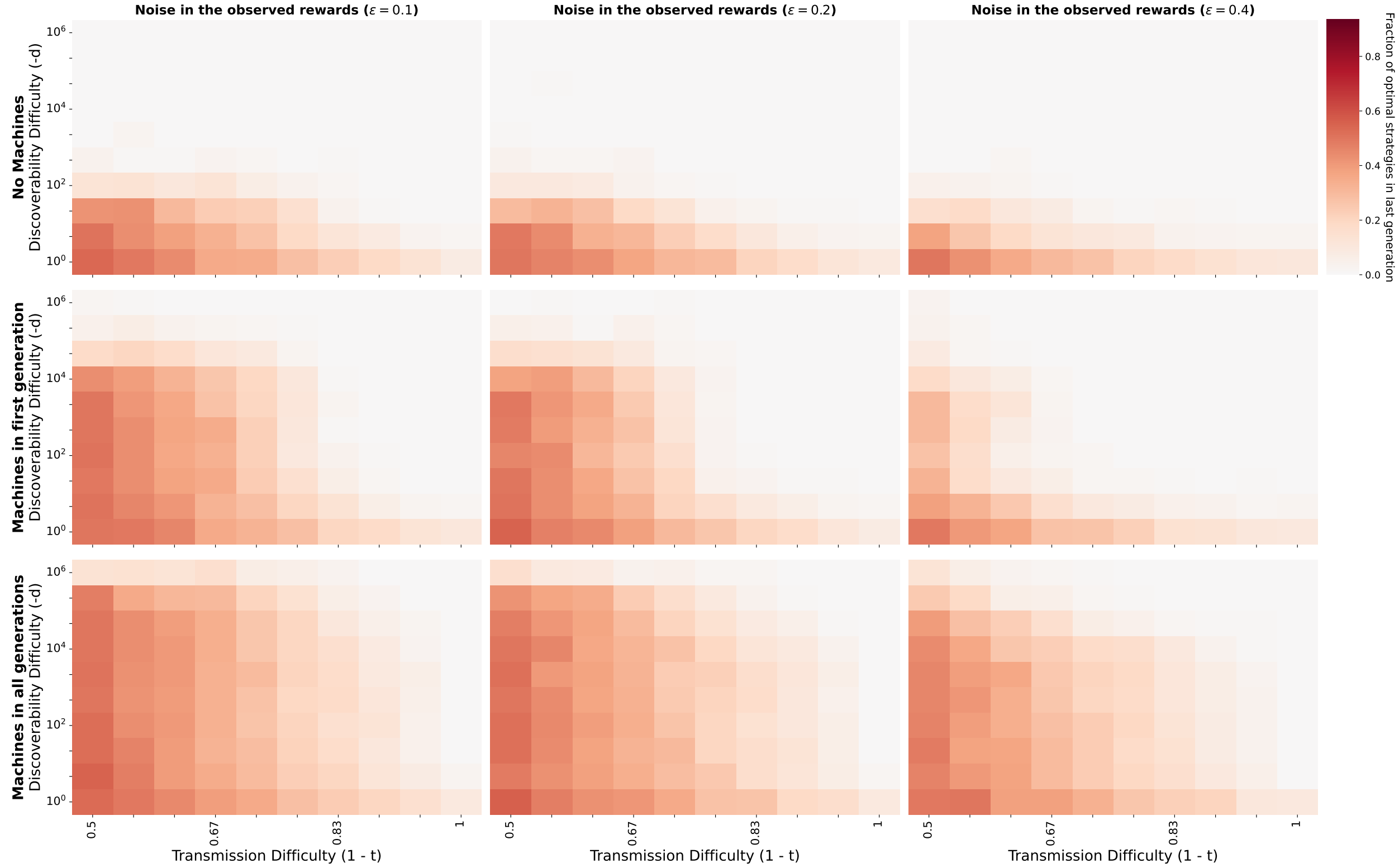

Figure S14: Selective social learning and machine availability. *We investigate how reward noise influences the adoption of the optimal solution under different machine-placement scenarios. Noise is introduced as independent Gaussian perturbations to individual realized rewards, with standard deviation* $\epsilon \in 0.1, 0.2, 0.4$*. We simulate 20 agents over 20 generations and compare scenarios with no machines, five machines in the first generation only, and five machines present in all generations. Machine advantage and bias are set to* $K_{\text{machine}} = 10000$ *and* $q_{\text{machine}} = 0.5$*, respectively. Color indicates the fraction of human agents adopting the optimal strategy in the final generation, with the color scale ranging from 0 (light) to 1 (dark). Results indicate that increasing reward noise degrades the reliability of performance-based teacher selection, hindering the intergenerational transmission of difficult-to-discover strategies, particularly when machine contributions are transient.*

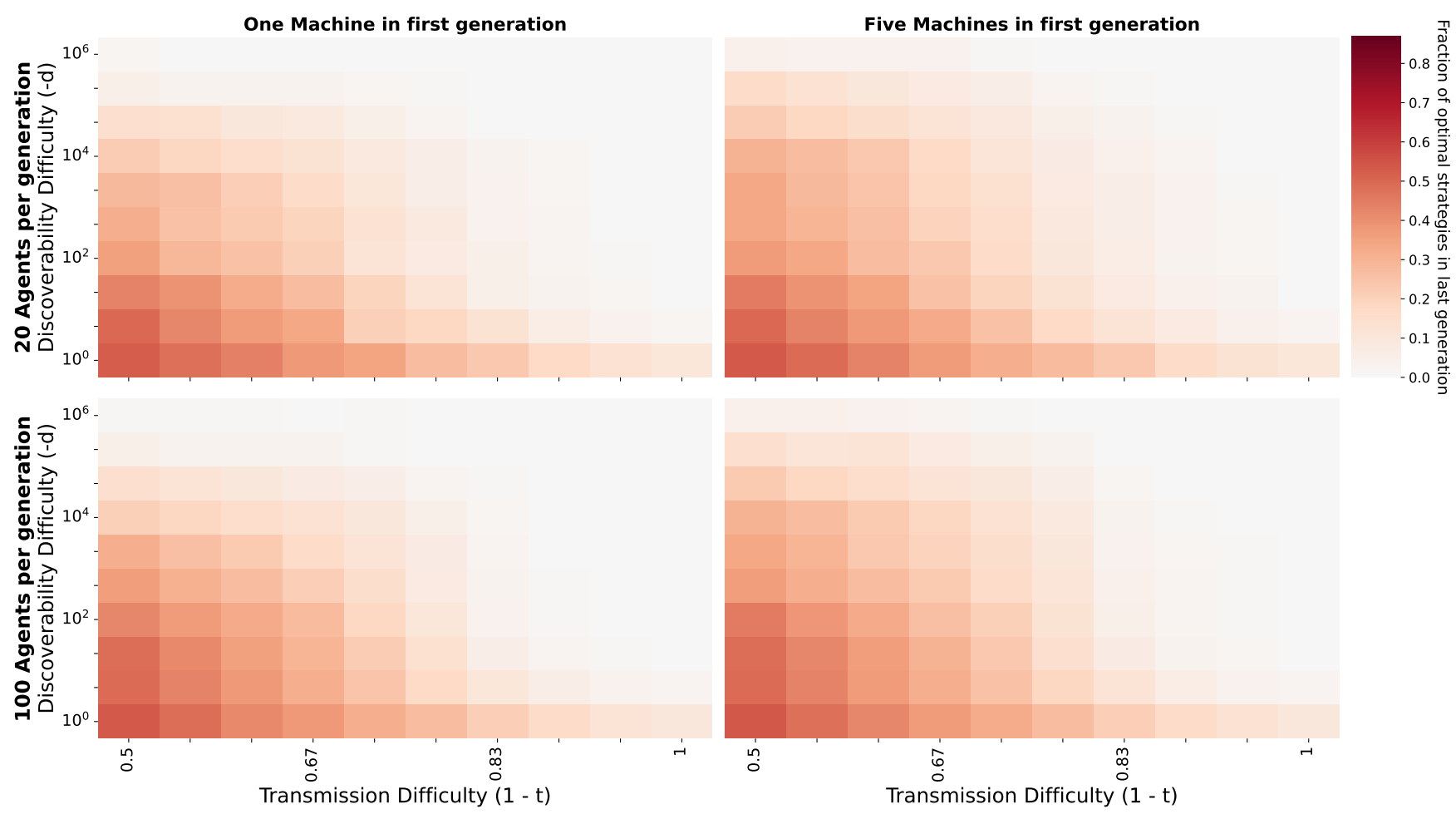

Figure S15: Robustness Analysis. *We examined the robustness of our results with respect to system size and the number of machines. Holding the social learning parameter (*$\tau = 0.1$*) and machine advantage (*$K_{\text{machine}} = 10000$ *and* $q_{\text{machine}} = 0.5$*) constant, we observed minimal sensitivity of the to variation in both the number of machines in the first generation and the number of agents per generation. Color indicates the fraction of human agents adopting the optimal strategy in the final generation, with the color scale ranging from 0 (light) to 1 (dark).*

	Prediction		
	1a	1b	2a
Condition = Human Population	−309.2	−229.1	−404.6
	(57.0)	(82.4)	(59.4)
	[−418.4, −200.1]	[−383.1, −55.5]	[−524.8, −286.1]
	$P < 0.001$	$P = 0.010$	$P < 0.001$
Generation	−46.5		
	(23.2)		
	[−93.2, −2.7]		
	$P = 0.054$		
Condition:Generation	86.4		
	(32.8)		
	[23.7, 146.8]		
	$P = 0.013$		
Intercept	1,843.5	1,814.8	1,886.5
	(40.3)	(58.3)	(42.0)
	[1763.5, 1918.2]	[1698.1, 1925.8]	[1806.6, 1975.1]
Observations	3,840	960	960
R^2_{marginal} / $R^2_{\text{conditional}}$	0.078 / 0.529	0.045 / 0.509	0.110 / 0.571

Table S1: *Model coefficients predicting task performance. Estimated coefficients with standard errors (SE), 95% bootstrap confidence intervals, and P-values (Satterthwaite approximation).*

	Prediction		
	1a	1b	2a
Condition = Human Population	−0.867	−0.644	−1.126
	(0.138)	(0.195)	(0.134)
	[−1.12, −0.61]	[−1.02, −0.25]	[−1.41, −0.88]
	$P < 0.001$	$P < 0.001$	$P < 0.001$
Generation	−0.152		
	(0.049)		
	[−0.25, −0.05]		
	$P = 0.002$		
Condition:Generation	0.201		
	(0.068)		
	[0.07, 0.33]		
	$P = 0.003$		
Intercept	1.209	1.054	1.396
	(0.098)	(0.139)	(0.097)
	[1.02, 1.40]	[0.78, 1.34]	[1.21, 1.59]
Observations	38,400	9,600	9,600
R^2_{marginal} / $R^2_{\text{conditional}}$	0.047 / 0.227	0.025 / 0.204	0.070 / 0.272

Table S2: *Model coefficients predicting behavioural congruency. Estimated coefficients (log-odds) with standard errors (SE), 95% bootstrap confidence intervals, and P-values (Wald z-test).*

	Prediction
	2b
Condition = Human Population	−4.180
	(0.617)
	[−6.30, −3.11]
	P < 0.001
Generation	−0.503
	(0.176)
	[−0.89, −0.10]
	P = 0.004
Condition:Generation	0.132
	(0.497)
	[−1.40,1.41]
	P = 0.609
Intercept	−0.578
	(0.281)
	[−1.18, 0.05]
Observations	960
R^2_{marginal} / $R^2_{\text{conditional}}$	0.539 / 0.670

Table S3: *Model coefficients predicting written strategy. Estimated coefficients (log-odds) with standard errors (SE), 95% bootstrap confidence intervals, and P-values (Wald z-test).*
