# Supplementary material for: Propagation and preservation of AI-discovered problem-solving strategies in human culture
Source: Nat Commun. 2026 Aug 18;17:8255. doi: 10.1038/s41467-026-76113-2 (PMC13487184; doi:10.1038/s41467-026-76113-2)
Supplement: Supplementary file 2 [file 41467_2026_76113_MOESM2_ESM.pdf]

nature portfolio

Corresponding author(s): Levin Brinkmann, Thomas Eisenmann, Iyad Rahwan

Last updated by author(s): 12/05/2026

Reporting Summary

Nature Portfolio wishes to improve the reproducibility of the work that we publish. This form provides structure for consistency and transparency in reporting. For further information on Nature Portfolio policies, see our Editorial Policies and the Editorial Policy Checklist.

Statistics

For all statistical analyses, confirm that the following items are present in the figure legend, table legend, main text, or Methods section.

n/a	Confirmed	
☐	☒	The exact sample size (*n*) for each experimental group/condition, given as a discrete number and unit of measurement
☐	☒	A statement on whether measurements were taken from distinct samples or whether the same sample was measured repeatedly
☐	☒	The statistical test(s) used AND whether they are one- or two-sided<br>*Only common tests should be described solely by name; describe more complex techniques in the Methods section.*
☐	☒	A description of all covariates tested
☐	☒	A description of any assumptions or corrections, such as tests of normality and adjustment for multiple comparisons
☐	☒	A full description of the statistical parameters including central tendency (e.g. means) or other basic estimates (e.g. regression coefficient) AND variation (e.g. standard deviation) or associated estimates of uncertainty (e.g. confidence intervals)
☐	☒	For null hypothesis testing, the test statistic (e.g. *F*, *t*, *r*) with confidence intervals, effect sizes, degrees of freedom and *P* value noted<br>*Give P values as exact values whenever suitable.*
☒	☐	For Bayesian analysis, information on the choice of priors and Markov chain Monte Carlo settings
☐	☒	For hierarchical and complex designs, identification of the appropriate level for tests and full reporting of outcomes
☐	☒	Estimates of effect sizes (e.g. Cohen's *d*, Pearson's *r*), indicating how they were calculated

Our web collection on statistics for biologists contains articles on many of the points above.

Software and code

Policy information about availability of computer code

Data collection	Data were collected through a custom-coded web app developed specifically for this experiment (React frontend, Flask backend). All code is available on Zenodo: https://doi.org/10.5281/zenodo.18547122
Data analysis	All statistical analyses were performed in R (version 4.3.2) using the lme4 package (version 1.1-35.1) for mixed-effects models. Bootstrap confidence intervals were computed in Python (NumPy). Analysis notebooks for visualisations and the agent-based simulation are also written in Python (pandas, matplotlib, seaborn). All analysis code is available on Zenodo: https://doi.org/10.5281/zenodo.18547122

For manuscripts utilizing custom algorithms or software that are central to the research but not yet described in published literature, software must be made available to editors and reviewers. We strongly encourage code deposition in a community repository (e.g. GitHub). See the Nature Portfolio guidelines for submitting code & software for further information.

Data

Policy information about availability of data

All manuscripts must include a data availability statement. This statement should provide the following information, where applicable:

- Accession codes, unique identifiers, or web links for publicly available datasets
- A description of any restrictions on data availability
- For clinical datasets or third party data, please ensure that the statement adheres to our policy

All experimental data are deposited on Zenodo (https://doi.org/10.5281/zenodo.18547122). The deposit contains both raw experimental data, as downloaded from

the experiment interface, and processed data with derived variables used in the analyses. All data are de-identified and provided at the individual-participant and move level.

Research involving human participants, their data, or biological material

Policy information about studies with human participants or human data. See also policy information about sex, gender (identity/presentation), and sexual orientation and race, ethnicity and racism.

Reporting on sex and gender	Sex/gender data were obtained from the Prolific platform for 1,272 participants (56.4% male, 43.2% female, 0.3% preferred not to say). Sex and gender were not analysed as variables, as they were not relevant to the research question on social learning. The study's findings are about general mechanisms of cultural transmission and are not expected to depend on sex or gender.
Reporting on race, ethnicity, or other socially relevant groupings	No data on race, ethnicity, or other socially salient categories were collected.
Population characteristics	Participants were aged 18 to 92 years (M = 39.67, SD = 12.28). 72.9% resided in the United Kingdom and 27.1% in the United States. All participants were proficient in English. Sex/gender distribution: 56.4% male, 43.2% female, 0.3% preferred not to say.
Recruitment	Participants were recruited through the online platform Prolific, screened for residence in the United States or the United Kingdom, English proficiency, and a Prolific approval rating of at least 95%. Recruitment via an online crowdsourcing platform may introduce self-selection bias toward individuals comfortable with online tasks.
Ethics oversight	Ethics Committee of the Max Planck Institute for Human Development (protocol A 2022-24). All participants provided informed consent prior to participation.

Note that full information on the approval of the study protocol must also be provided in the manuscript.

Field-specific reporting

Please select the one below that is the best fit for your research. If you are not sure, read the appropriate sections before making your selection.

☐ Life sciences ☒ Behavioural & social sciences ☐ Ecological, evolutionary & environmental sciences

For a reference copy of the document with all sections, see nature.com/documents/nr-reporting-summary-flat.pdf

Behavioural & social sciences study design

All studies must disclose on these points even when the disclosure is negative.

Study description	quantitative experimental study
Research sample	Participants were recruited through the online platform Prolific, while screening for individuals residing in the United States and the United Kingdom, being proficient in English, and maintaining an approval rating of at least 95%.
Sampling strategy	Online convenience sample (see above). We determined the goal of 15 trees per condition by simulating data via an agent-based model.
Data collection	Data were collected through a web app developed specifically for this experiment.
Timing	Data were collected between 19 and 20 February 2024.
Data exclusions	As preregistered, participants were excluded based on two criteria: 1) Participants who took more than 25 minutes, deemed unreasonably long; 2) Participants with missing moves in any of their demonstration trials, as subsequent generations could not replicate incomplete solutions. These criteria also implicitly served as an attention check. Of the 1,303 participants who completed the experiment, 148 were excluded under these conditions. Their spots were then filled by newly recruited participants. No other exclusions were made.
Non-participation	Due to the strict exclusion criteria, no additional cases of non-participation were recorded.
Randomization	Participants were dynamically assigned to a random available position in the populations, prioritizing the completion of existing populations before adding participants to new ones.

Reporting for specific materials, systems and methods

We require information from authors about some types of materials, experimental systems and methods used in many studies. Here, indicate whether each material, system or method listed is relevant to your study. If you are not sure if a list item applies to your research, read the appropriate section before selecting a response.

Materials & experimental systems

n/a	Involved in the study
☒	☐ Antibodies
☒	☐ Eukaryotic cell lines
☒	☐ Palaeontology and archaeology
☒	☐ Animals and other organisms
☒	☐ Clinical data
☒	☐ Dual use research of concern
☒	☐ Plants

Methods

n/a	Involved in the study
☒	☐ ChIP-seq
☒	☐ Flow cytometry
☒	☐ MRI-based neuroimaging

Plants

Seed stocks	NA
Novel plant genotypes	NA
Authentication	NA
