# Supplementary material for: Propagation and preservation of AI-discovered problem-solving strategies in human culture
Source: Nat Commun. 2026 Aug 18;17:8255. doi: 10.1038/s41467-026-76113-2 (PMC13487184; doi:10.1038/s41467-026-76113-2)
Supplement: Supplementary file 3 [file 41467_2026_76113_MOESM3_ESM.pdf]

nature portfolio

Peer Review File

Propagation and preservation of AI-discovered problem-solving strategies in human culture

Corresponding Author: Dr Levin Brinkmann

This file contains all reviewer reports in order by version, followed by all author rebuttals in order by version.

Version 0:

Reviewer comments:

Reviewer #1

(Remarks to the Author)
This paper investigates the conditions under which machine-discovered strategies can be adopted and preserved within human cultural evolution. The authors combine a multi-generational social-learning experiment with agent-based simulations to test three hypothesised requirements for persistent “machine discoveries”: appropriate discovery difficulty, low transmission difficulty, and a recognizable selective advantage. In their laboratory task, deep-RL agents reliably discovered an “optimal” reward-network strategy that human participants, owing to their myopic loss-avoidance bias, almost never discovered themselves. Yet once this machine-generated strategy was introduced, it spread across human generations and remained detectable long after the machine players were gone. Simulations further explored the parameter space of discovery and transmission difficulty and confirmed that machine contributions can durably expand the human cultural repertoire. Overall, this is a very interesting and timely study, we do however have some points:

Major comments
1/
The experiment hinges on the idea that humans fail to discover the optimal strategy because of an inherent cognitive biases (such as prioritising immediate reward at the expense of long-term gains) whereas machines, unconstrained by such biases, can discover “alien” strategies. Yet humans were given five minutes to train on six networks, while the RL agents trained on 1,000 networks in the same time. Is human failure driven by a constitutive cognitive bias or simply by limited training opportunities? The authors should discuss or attempt to disentangle these two possibilities.
2/
Relatedly, what is the minimal number of reward network trials needed for the AI agent to reach an optimal policy? How many trials (or minutes) would human participants need to reliably discover the same strategy? If humans were given, say, 15 minutes, would machine discovery still look “alien,” or would the advantage disappear? Because the paper frames its contribution in analogy with Go and chess—where machines uncover strategies that humans have failed to find after centuries of play—this distinction is critical.
3/
In Experiment 1 (“Humans Preserve the Machine Discovery”), the authors report that in human-machine populations the optimal strategy persisted across generations and all final-generation players were descendants of a machine demonstrator. But the data mainly show that the reward-maximising strategy, whoever first discovers it, is preserved. The fact that machines find it more often seems due to their computational speed and the imposed time constraints, not to any uniquely “machine-like” quality of the strategy itself. This suggests that what is transmitted is simply the correct strategy, not necessarily a strategy with distinctive machine properties. Moreover, in 6 of 15 mixed populations the strategy eventually disappeared despite identical machine discovery processes. This pattern looks compatible with chance rather than any inherent “machine insight.” The authors should clarify whether they see evidence for a qualitatively distinct machine contribution beyond mere discovery frequency.
4/
The description of Figure 5 is difficult to follow. As presented, panel A appears to show a non-linear relationship in which higher transmission difficulty somehow predicts lower discovery difficulty. Please explain more clearly what the axes and boundaries represent and how to interpret the apparent non-linearity.
5/
Panel B is said to show the “boost in average reward of mixed populations over human-only populations in the final generation.” Presumably this heatmap represents human reward minus human-machine reward. Given the theoretical

framing, one might expect the machine advantage to grow when discovery or transmission difficulty is high, i.e. precisely where machines should help most. Instead, the advantage appears bounded within moderate, human-achievable levels of difficulty. Could this indicate a limitation of the current simulation assumptions? Some discussion would be welcome.
6/
The three proposed conditions for machine discoveries to be culturally adopted—appropriate discovery difficulty, low transmission difficulty and recognizable selective advantage—are compelling, but their theoretical origin is not clearly explained. Please clarify why these conditions should be regarded as necessary (and perhaps sufficient) rather than merely plausible.
7/
The manuscript's structure blends introduction, design and results in a less conventional way; if this is intentional or aligned with the journal's style, discard this point
Minor comments

• When reporting regression results (of any type), please include p-values or at least a measure of model comparison or fit. Even if very large betas are almost certainly significant, it is preferable to document this explicitly.

• The simulation-based power analysis is only briefly described. Please expand on how it was performed, especially if pilot data informed the parameter choices.

• The procedure by which participants "recorded themselves" for future generations is not entirely clear. Did they write textual descriptions, use webcams, or something else? If written, did you examine whether surface features (e.g., spelling errors, verbosity) affected the likelihood that a strategy was preserved? How did the machine players produce their recorded demonstrations?

• The paper would benefit from a brief limitations section (with or without a dedicated header) to acknowledge, for example, the constrained laboratory setting and the potential confound between cognitive bias and training time.

(Remarks on code availability)

Reviewer #2

(Remarks to the Author)
The article investigates the conditions under which machine-induced cultural shifts occur using controlled behavioral experiments and computer simulations. The study makes an important contribution by approaching the topic of cultural evolution with AI (simple reinforcement-learning agents). It is technically commendable that the authors conducted an experiment requiring about 40 participants per group (8 participants × 5 generations, in the human-population condition). I find this study meaningful.
While I have no concerns regarding the importance of the research question, there are several points the authors may wish to address to improve the manuscript regarding the analysis and interpretation that should be improved. Please find some comments and questions below. Because the document does not include line numbers, some of the following comments may be unclear regarding which parts are being discussed. I would appreciate it if the authors could include line numbers in the files when they have the opportunity to revise and resubmit the article.

1. There is a gap between what the authors state in the abstract and introduction and what they test in their experiment. The authors suggest that three conditions are involved in machine-induced cultural shift based on cultural evolution theory: Appropriate Discovery Difficulty, Low Transmission Difficulty, and Recognizable Selective Advantage. In the abstract, they state, "Using a cultural transmission experiment and an agent-based simulation, we demonstrate that when these conditions are met, machine-discovered strategies can be transmitted, understood, and preserved by human populations, leading to enduring cultural shifts." However, these conditions are not manipulated in the experiment presented in the paper (the preregistration and the discussion mention pilot data, but from what I can see, it is not publicly available in the main paper). Of course, it makes sense to test the three conditions in a low-cost simulation and then test just one specific combination in a more expensive experiment. However, since a simulation and an experiment are not the same, what is demonstrated in the simulation has not been demonstrated in the experiment. Therefore, it may be inappropriate to state in the abstract and introduction that they have shown the importance of the three conditions using both the experiment "and" the simulation. Conversely, if they were to argue that the experiment and simulation are essentially the same, then the experiment would have been unnecessary, and the simulation alone would have sufficed.

2. In Figure 4, the dots represent individual populations (15 populations per condition), but the analysis reported in reference to Figure 4 seems to use each participant as the unit of analysis, which is misleading.

3. In Figure 4, the authors might want to include standard errors or CIs around the means.

4. In the preregistration, four models (1a, 1b, 2a, and 2b) were listed. The authors should clarify which of these models corresponds to the analysis they describe in the main text when they state, "Our preregistered linear mixed effects model on these latter generations supported that this difference was significant." Furthermore, if they used model 1a, they should

explicitly state that it is a model that tests for an interaction. If they are reporting a model that does not include the interaction, they should also report the model that does include the interaction, as specified in their preregistration.

5. If individual participants are the unit of analysis, the model must explicitly account for the nesting of participants within trees and generations. Otherwise, there is a risk of a Type I error. Unfortunately, the preregistered models do not seem to include this hierarchical structure. I would recommend that the authors re-analyze their data with the appropriate nesting structures. If the model fails to converge due to its complexity, it suggests that the sample size was too small to begin with, and it is not a valid reason to ignore the hierarchy in the analysis.

6. In the discussion, the authors claim, “Our pilot experiments illustrated the key challenge of achieving the right balance between discovery difficulty and transmissibility.” If the authors wish to maintain this claim, they should report the pilot data publicly.

(Remarks on code availability)

Reviewer #3

(Remarks to the Author)
This paper examines the conditions under which machines can become drivers of (persistent) cultural change. It studies cultural evolution in populations of humans trying to solve a problem, vs populations of humans and machines; and tries to determine what conditions are necessary for machine-discovered ideas to spread *and* be fully incorporated by humans. It identifies three such conditions for solutions: they must have a selective advantage, they must be non-trivial, and they must be learnable. This is done with cultural transmission experiments, that are nicely combined with computer simulations in order to examine counterfactuals and establish these conditions are all necessary.

For the experiment, a specific task is designed such that humans have a bias that makes it hard to discover optimal solutions. Machines, on the other hand, can quickly find optimal solutions using standard learning algorithms. Around ~1.2k participants were split into 30 populations; half were Human-Only, and half were Human-Machine, including some “machine players” inserted in Generation 0. After practicing solutions, participants chose a demonstrator from five candidates shown with their average scores (without knowing who was human or machine), watched that replay, reproduced it, and finally solved new networks and recorded their own demonstration for the next generation. This design allows the authors to test whether an machine-discovered strategy would be learned by humans and preserved across generations even in the absence of machines.

The experiment is complemented with some agent-based simulations that mirror the lab setting. Here, each agent can pick a strategy (random, myopic, or optimal), and in each generation it either explored on its own or learned socially by picking a demonstrator from five candidates in the previous generation. The simulation allows control over discovery and transmission difficulty. The key result (Figure 5, p. 11) is that machines create a lasting boost only when the optimal strategy is hard for humans to discover but still easy to learn; if discovery is easy, learning is random, or transmission is too difficult, the gains provided by machine discoveries are small.

Some minor comments:

- I think it would be interesting to tease out more when each condition is required. The fact that selective advantage is necessary but not sufficient is somehow puzzling. I suppose this has to do not only with ideas spreading, but the fact that they need to be incorporated by humans in the absence of machines. If that is the case, would machines teaching each other will again guarantee that selective advantage on its own is enough? I feel this could be spelled out more clearly in the discussion.

- Can the authors explain the appeal of the random strategy in the simulation? Would the same results arise without it?

- Can the authors comment on what they would expect with tasks that are more fair or where the advantage of machines is large but not huge? The task is particularly easy to solve for machines: not even Q-learning would be required for an optimal solution to be found.

This is I think a great paper that combines a fantastic question with good methods. The topic is fascinating and will be of interest to computer scientists and social scientists alike. Overall, the paper convincingly shows that a machine-discovered, non-trivial but learnable strategies can spread and persist. It would be good to extend these to other less engineered tasks where the advantage of machines is less salient, but this may be part of the many extensions I am sure the work will inspire.

I recommend publication.

(Remarks on code availability)
Code is complete and seems to be of high standard.

Reviewer #4

(Remarks to the Author)
This paper is a good, straightforward demonstration of what it sets out to do. The authors show that machines can discover solutions that are counterintuitive to humans, and that humans can adopt them. The experiment and the model seem solid, well executed, and clearly presented. I thought the fact that the agent-based model recovers the theoretical expectations presented in the introduction a nice touch. I don't find the fact that humans can recognise good solutions and use them particularly surprising, and I don't think it is my place to comment on whether the novelty of this result qualifies the paper for publication in Nature Communications. Below I summarise some concerns I have with the framing, and some suggestions for improving clarity.

One niggling question I have is how much the dynamic captured here is about machines specifically vs. humans being able to recognise and integrate new information in their strategies, and whether this difference even matters. If I read the results correctly (Fig. 3, S6), one human participant discovered the optimal strategy and passed it on to the next generation, but this took place at the end of the experiment (generations 3-4) so we don't know if this strategy would have persisted further. The expectation is that it would have. Would the human participants have discovered the optimal solution more often with enough time (6 trials seems low)? The novel point is then not that machines can transform human culture, but that machines can generate new solutions that humans can readily use.

Another point that I think should be discussed directly in the paper is the role that payoff-bias (i.e. the fact that humans have access to score and can use score to dictate their social learning) plays for these results. I am assuming that participants chose the highest-scoring model most of the time (it would be nice to see some numbers on this), which again means humans have built-in mechanisms to take advantage of good information and good solutions. The phrasing in the results section is potentially misleading. The section titled "Humans Preserve the Machine Discovery" seems to imply there is something special about the machine solution that ensures it is preserved, and I suspect the something special is better score. I suggest the authors mention this directly in the text (or better yet, run additional analyses looking at the relationship between score and transmission). Similarly, the next section "Humans are Behaviourally Congruent with the Machines" seems to suggest the human participant intend to copy the machines – would you find the same congruence with the loss strategy discovered by the one human participant in the human-only condition?

Minor comments below:
• On page 7 please mention how many training trials generation 0 humans get, and mention how many individual learning trails generations 1-4 get and whether is it before/after social learning
• Page 9 – "Panel B in Figure 4 shows the proportion of human moves congruent with machine-generated moves, summarized by generation and population." – which machine generated moves?
• Fig. 4c – why do human-only participants in generation 0 mention a loss strategy and how did this translate into the participants' score?
• Figure 5a – I struggled to parse this description, consider adding more detail
• Page 12 – in the discussion on population size and complex solutions, I would argue that the kind of phenomenon described here might benefit smaller populations more. Larger populations might have a higher chance of discovering the optimal solution, which would make the machine intervention redundant. It all depends on how novel/outside the human range the machine-discovered solutions can be.
• Sentence "Our results apply particularly to scenarios in which human exploration is constrained by time rather than computational cost, machine exploration remains scalable" on page 14 might need editing
• The choices of parameter values for the agent-based model require justification beyond the fact that they nicely produced the curve in Fig. 2b – why the chosen d and t values, why was the discovery rate for machine agents set to 1000 higher than human agents, what does superior mean in "Upon discovering a new strategy, the agent updated its preferred strategy if the new strategy was superior to the previous one."

(Remarks on code availability)

Reviewer #5

(Remarks to the Author)
I co-reviewed this manuscript with one of the reviewers who provided the listed reports. This is part of the Nature Communications initiative to facilitate training in peer review and to provide appropriate recognition for Early Career Researchers who co-review manuscripts.

(Remarks on code availability)
I co-reviewed this manuscript with one of the reviewers who provided the listed reports. This is part of the Nature Communications initiative to facilitate training in peer review and to provide appropriate recognition for Early Career Researchers who co-review manuscripts

Version 1:

Reviewer comments:

Reviewer #1

(Remarks to the Author)
The authors successfully addressed our suggestions.

(Remarks on code availability)

Reviewer #2

(Remarks to the Author)
The authors have appropriately addressed my comment. I appreciate their careful and thorough re-analysis of the statistical issues.

(Remarks on code availability)

Reviewer #3

(Remarks to the Author)
I am satisfied with the current version, and have enjoyed the discussion arising from the review process. The authors have clearly addressed all the comments and engaged with all the questions; as a result the paper is even better than it already was.

(Remarks on code availability)
Code is of high standard.

Reviewer #4

(Remarks to the Author)
I'm happy with the changes made in response to my comments, and recommend publication.

(Remarks on code availability)

Reviewer #5

(Remarks to the Author)
I co-reviewed this manuscript with one of the reviewers who provided the listed reports. This is part of the Nature Communications initiative to facilitate training in peer review and to provide appropriate recognition for Early Career Researchers who co-review manuscripts.

(Remarks on code availability)

Open Access This Peer Review File is licensed under a Creative Commons Attribution 4.0 International License, which permits use, sharing, adaptation, distribution and reproduction in any medium or format, as long as you give appropriate credit to the original author(s) and the source, provide a link to the Creative Commons license, and indicate if changes were made.
In cases where reviewers are anonymous, credit should be given to 'Anonymous Referee' and the source.
The images or other third party material in this Peer Review File are included in the article's Creative Commons license, unless indicated otherwise in a credit line to the material. If material is not included in the article's Creative Commons license and your intended use is not permitted by statutory regulation or exceeds the permitted use, you will need to obtain permission directly from the copyright holder.
To view a copy of this license, visit https://creativecommons.org/licenses/by/4.0/

We thank the reviewers for their careful and constructive evaluation of our work, which allowed us to substantially strengthen the manuscript.

We have addressed all major and minor comments in detail in the item-by-item response below. A revised version of the manuscript with tracked changes is provided as a Related Manuscript File.

Reviewer #1 (Remarks to the Author):

This paper investigates the conditions under which machine-discovered strategies can be adopted and preserved within human cultural evolution. The authors combine a multi-generational social-learning experiment with agent-based simulations to test three hypothesised requirements for persistent "machine discoveries": appropriate discovery difficulty, low transmission difficulty, and a recognizable selective advantage. In their laboratory task, deep-RL agents reliably discovered an "optimal" reward-network strategy that human participants, owing to their myopic loss-avoidance bias, almost never discovered themselves. Yet once this machine-generated strategy was introduced, it spread across human generations and remained detectable long after the machine players were gone. Simulations further explored the parameter space of discovery and transmission difficulty and confirmed that machine contributions can durably expand the human cultural repertoire. Overall, this is a very interesting and timely study, we do however have some points:

We thank the reviewer for their thoughtful evaluation of our work and are pleased that they find the study interesting and timely. We address their points one by one below.

Major comments

1/

The experiment hinges on the idea that humans fail to discover the optimal strategy because of an inherent cognitive biases (such as prioritising immediate reward at the expense of long-term gains) whereas machines, unconstrained by such biases, can discover "alien" strategies. Yet humans were given five minutes to train on six networks, while the RL agents trained on 1,000 networks in the same time. Is human failure driven by a constitutive cognitive bias or simply by limited training opportunities? The authors should discuss or attempt to disentangle these two possibilities.

We are grateful to the reviewer for this insightful question, which allows us to examine in greater depth the interaction between bias and training opportunities. In response, we have added a new analysis to the manuscript (see Supplementary Text, *Estimating Human Exploration Bias*) that investigates human exploration bias in these tasks.

We show that humans exhibit substantial systematic bias in their exploration relative to a random baseline. Specifically, participants used significantly fewer paths involving losses and, consequently, reached level-3 nodes at a significantly lower rate. We further compared participants in generation 0, who were endowed with six individual learning trials, with participants in later generations, who completed only two individual learning trials followed by four social learning trials. We found no evidence of a difference in exploration bias

between these groups. Instead, across successive trials, participants reduced their likelihood of incurring large losses.

In addition, we disentangled bias and learning opportunities in the agent-based simulations, which were previously subsumed within a single parameter. We now present additional simulations demonstrating that both reduced bias and increased learning opportunities can independently give rise to the observed machine advantages, with the strongest effects emerging when these factors coincide (see updated Figure 5 and new Figure S12).

In this study, we designed the machine to explore strategies at random. Although experimentally constrained in the present work, the framework of computational rationality suggests that this strategy is adaptive when exploration costs are negligible. An agent with a larger exploration budget can afford to rely less on priors during learning and to adopt a lower learning rate, thereby sampling regions of the strategies space that appear unlikely to contain successful strategies.

Accordingly, in the revised manuscript, we clearly demonstrate that our analysis targets a regime in which both human bias and temporal constraints limit exploration. We show numerically that, in principle, either factor alone can be sufficient to generate a machine advantage, while the strongest effects are expected when both operate in combination.

Specific changes:

- **Completely new analysis in SI**: Supplementary Text, Estimating Human Exploration Bias
- **Significantly Updated Agent-Based Model: See method and result section**
- **Addition to the introduction**: *This difference in resource constraints allows machines to explore much larger hypothesis and action spaces, reducing reliance on strong priors and heuristics that shape human search and enabling the discovery of strategies outside the region typically accessible to human exploration.*
- **Addition to the result section**: *While the deep Q-learning agent, through extensive exploration over 20000 epochs across 1000 networks, discovered and reliably reproduced the optimal strategy within less than five minutes (Supplementary Fig.S7), human participants, by contrast, exhibited a systematic avoidance of loss-incurring trajectories: paths involving three losses were strongly under-sampled relative to a matched random baseline (LOR=-1.22, CI95%=[-1.38,-1.06]), substantially reducing the likelihood of discovering the optimal strategy (see also Supplementary Text).*
- **Updated agent-based model:** *We first confirmed that, in homogeneous populations and under high social learning success rates, discovery is constrained by bias q, limited exploratory capacity K, and the number of generations elapsed, G (see Supplementary Figure S12). We then focused on scenarios with heterogeneous exploration capacity and bias, in which the majority of agents (i.e., human agents) are limited in their exploration capacity k_m = 10 and exhibit a strong bias q_m = 0.01 toward myopic solutions. We systematically manipulated the exploratory capacity and bias of five machine agents added to the first generation and examined the resulting adoption of optimal strategy by human agents in the final generation. Increasing the exploratory capacity K_m in the range 1000 to 100000 of the machine*

agents and neutralizing their bias toward myopic strategies (q_m = 0.5) both increased the likelihood of discovering the optimal strategy. Consequently, introducing such machine agents enabled populations to adopt optimal strategies even under high discovery difficulty and thus expanded the horizon of obtainable strategies within a population.

- **New / Updated Figures**: Main Figure 5, Supplementary Figure S12
- **Clarification in the discussion:** *In our experiment, the state space was small enough to permit brute-force exploration, and the primary role of Q-learning was to extract a generalizable, model-free strategy from this experience. In many real-world domains, however, solution spaces are intractable and exploration is resource-intensive.*
- **New in the discussion:** *As a controlled online experiment, our design necessarily reflects stylized conditions, with individual learning constrained to brief exploration periods that may differ from real-world settings. However, analyses of participants in generation 0 with greater learning opportunities provide no evidence that extended exploration budgets attenuate loss-related biases (see Supplementary Text).*

2/

Relatedly, what is the minimal number of reward network trials needed for the AI agent to reach an optimal policy? How many trials (or minutes) would human participants need to reliably discover the same strategy? If humans were given, say, 15 minutes, would machine discovery still look "alien," or would the advantage disappear? Because the paper frames its contribution in analogy with Go and chess—where machines uncover strategies that humans have failed to find after centuries of play—this distinction is critical.

The RL agent was trained for 20,000 epochs across 1,000 networks. We now report these numbers more prominently in the main manuscript. Approximately half of this time was required for the first algorithm to discover the optimal strategy (see Fig. S7). Note, however, that no effort was invested in optimising the learning speed of the algorithm. The primary objective was to assess whether discovery within a timeframe comparable to that of human participants in the experiment was possible. As the algorithm readily achieved this goal, no further optimisation was pursued.

We agree with the reviewer in the implicit assumption that, given enough time, a single individual could in principle discover and master the optimal strategy. Likewise, in many laboratory studies of cumulative cultural evolution, individual learners can benefit from learning-curve effects and the absence of transmission noise, and may therefore outperform a multi-generational group when total experience is equalized (Derex et al., 2024, https://doi.org/10.1073/pnas.2322886121). However, the aim of cumulative cultural evolution research is not to demonstrate that populations necessarily outperform individuals given the same amount of time. Rather, the central phenomenon is that culture allows experience to be accumulated beyond the limits of a single lifetime, thereby enabling strategies to be adopted that no individual could realistically discover on their own.

A similar asymmetry applies to machines. AI systems can explore at speeds and scales that exceed human capacities by orders of magnitude, reaching an exploration budget that no human could attain within a lifetime. It is precisely this inequality in feasible exploration that our study targets. In the case of AlphaGo, for instance, the system played on the order of a

million self-play games. It is plausible that a human brain could in principle arrive at comparable strategies if such an amount of exploration were possible, but this is clearly infeasible for any individual. Humans can only accumulate such experience culturally, at the cost of transmission noise and the need for successive generations to relearn and reconstruct knowledge. Our experiments are designed to examine the impact of machines under these constraints.

Specific Changes:

- **Specification of assumption in the introduction**: *Experimental work has yet to investigate how <u>computational heterogeneity and asymmetric exploration capacities</u> shape human–machine societies, thereby extending prior work, which typically considers only one agent type [15, 22, 23].*
- **Addition to the Results**: *While the deep Q-learning agent, through extensive exploration over 20000 epochs across 1000 networks, discovered and reliably reproduced the optimal strategy within less than five minutes (Supplementary Fig.S7),...*
- **Clarification in the Discussion:** *In our experiment, exploration at <u>multiple orders of magnitude greater capacity</u> allowed machine players to discover a superior strategy that offset human cognitive biases toward myopic and suboptimal strategies and, as such, was effectively beyond human reach <u>within the constraints exposed by the experiment</u>. This does not mean that the optimal strategy was impossible to discover for human participants at all, but <u>limitations in time and cognitive biases</u> [1] made it exceedingly rare.*
- **New to the Discussion**: *Human culture supports the cumulative accumulation of knowledge and skills across generations, enabling individuals to benefit from collective experience that far exceeds what any single person could acquire in isolation [9, 10]. Laboratory studies explicitly compressing generational timescales into hours or minutes show that social transmission can overcome time constraints and the costs of individual exploration [36, 37]. Building on this literature, the present work suggests that machines may function as novel sources of cumulative cultural input. By learning from machines, humans may adopt and transmit hard-to-conceive optimal strategies, thereby expanding their cognitive repertoire without incurring the cognitive costs and time typically associated with discovering such strategies. Our results apply particularly to scenarios in which human exploration is constrained, while machine exploration remains scalable.*

3/
In Experiment 1 ("Humans Preserve the Machine Discovery"), the authors report that in human-machine populations the optimal strategy persisted across generations and all final-generation players were descendants of a machine demonstrator. But the data mainly show that the reward-maximising strategy, whoever first discovers it, is preserved. The fact that machines find it more often seems due to their computational speed and the imposed time constraints, not to any uniquely "machine-like" quality of the strategy itself. This suggests that what is transmitted is simply the correct strategy, not necessarily a strategy with distinctive machine properties. Moreover, in 6 of 15 mixed populations the strategy eventually disappeared despite identical machine discovery processes. This pattern looks compatible with chance rather than any inherent "machine insight." The authors should

clarify whether they see evidence for a qualitatively distinct machine contribution beyond mere discovery frequency.

We thank the reviewer for this important clarification and agree that greater precision is needed regarding what constitutes a "machine" contribution.

First, we like to clarify that a strategy being machine-like (or "alien," as described elsewhere) is less a property of the machine that discovers it than a reflection of humans' failure to discover it. There can be multiple reasons why humans fail to identify strategies that machines are able to uncover. Drawing on the existing literature and the results of our experiment, we propose at least two canonical sources of such failure: limited exploration budgets due to time and speed constraints, and systematic exploration biases (whether inertial or culturally shaped). In this revision, we therefore expand the Discussion to unpack in greater detail how these two mechanisms can differentially shape human and machine discovery processes.

Second, the fact that in some populations the strategy eventually went extinct is a signature of the cultural transmission process itself. In line with standard models of cumulative culture, rare and counterintuitive innovations are vulnerable to stochastic loss under copying noise and finite population size. Our simulations show that even when a strategy has a genuine selective advantage, its long-term survival is probabilistic and depends on transmission fidelity and population structure. The experiment thus demonstrates not an inevitability, but the possibility that machine-discovered strategies, initially outside the human discovery horizon, can enter a cultural lineage and persist for multiple generations.

Specific Changes:

- **We completely redesigned Figure 1B:** In the new version, we explicitly highlight increased computational capacity and reduced bias as the key mechanisms for machines to discover new strategies.
- **Addition to the introduction (as stated above)**: *This difference in resource constraints allows machines to explore much larger hypothesis and action spaces, reducing reliance on strong priors and heuristics that shape human search and enabling the discovery of strategies outside the region typically accessible to human exploration [21].*
- **Clarification in the introduction:** *This may lead machines to discover strategies that are <u>unknown and potentially even counterintuitive</u> to humans yet have superior performance (see Figure 1B, vertical axis).*
- **Clarification in the introduction:** *Meanwhile, we expected that a few minutes of training on our task would be sufficient for a deep-Q-learning algorithm (the "machine player") to successfully discover the optimal strategy <u>through unbiased exploration at scale</u>.*
- **Addition to the Discussion:** *<u>The results presented in this work suggest that scale of exploration alone can enable machines to discover strategies that humans fail to uncover, but are able to learn. They also indicate an interplay between exploration scale and bias, or cultural entrenchment, that constrains human exploration. Building on this insight,</u> future studies could explore how the enhanced exploratory depth and*

breadth enabled by machines might help overcome structural limitations, such as entrenched path dependencies and biased expectations shaped by a historically conditioned epistemic horizon [47, 29].

- **Addition to the Discussion:** *Despite most populations maintaining the algorithmically discovered optimal strategy, the results show that even selectively advantageous strategies remain vulnerable to stochastic loss under finite populations and transmission noise.*

4/
The description of Figure 5 is difficult to follow. As presented, panel A appears to show a non-linear relationship in which higher transmission difficulty somehow predicts lower discovery difficulty. Please explain more clearly what the axes and boundaries represent and how to interpret the apparent non-linearity.

Thank you for this question. Upon revisiting the manuscript, we realized that this figure was indeed challenging to interpret. In the revised version, we present an updated set of figures that, in our view, more clearly convey the main message of the paper.

In particular, we updated Figure 1B. The earlier version was directly based on our simulation results. However, as the reviewer correctly noted, this representation raised questions that the manuscript could not adequately address. We therefore now present a more stylized version of the figure, which allows us to more clearly describe the different regimes and mechanisms at play.

We likewise revised Figure 5 in response to the reviewer's feedback. In the updated version, we directly depict the adoption rate of hybrid populations as a function of the capacity and behavior of added superhuman agents. Accordingly, we moved the manipulation of the social learning mechanism to the Supplementary Material.

Although this relationship is no longer central to the revised manuscript, we would like to briefly clarify the origin of the previously observed non-linearity. A population can sustain a high-performing strategy when the probability of rediscovering the strategy in a given generation is equal to or greater than the probability of collectively losing it through imperfect transmission. This balance gives rise to a non-linear relationship in which the persistence of the optimal strategies depends on both transmission difficulty and discovery difficulty in a non-trivial manner.

Specific Changes:

- Updated Figure 1.B
- Updated Figure 5
- Updated Figure S13

5/
Panel B is said to show the "boost in average reward of mixed populations over human-only populations in the final generation." Presumably this heatmap represents human reward minus human-machine reward. Given the theoretical framing, one might expect the machine

advantage to grow when discovery or transmission difficulty is high, i.e. precisely where machines should help most. Instead, the advantage appears bounded within moderate, human-achievable levels of difficulty. Could this indicate a limitation of the current simulation assumptions? Some discussion would be welcome.

The model assumes that machines, although more powerful in exploration than humans, are nevertheless subject to certain limits. As a result, our earlier results showed no advantage for hybrid populations in regimes where even machine agents fail to discover effective strategies. In the revised manuscript, we make this assumption substantially more transparent.

In particular, we revised the conceptual Figure 1B to explicitly illustrate these limits. We also updated Figure 5 to directly manipulate both the exploration capacity of machine agents and their behavioral bias. This revised representation, developed in response to the reviewer's comments, makes the underlying modeling assumptions considerably more explicit.

The updated figures show that more powerful machine agents can indeed enable humans to adopt strategies that are more difficult to discover. However, limitations in transmissibility continue to constrain long-term preservation, even in these regimes.

Specific Changes:

- Updated Figure 1.B
- Updated Figure 5

6/
The three proposed conditions for machine discoveries to be culturally adopted—appropriate discovery difficulty, low transmission difficulty and recognizable selective advantage—are compelling, but their theoretical origin is not clearly explained. Please clarify why these conditions should be regarded as necessary (and perhaps sufficient) rather than merely plausible.

The dimensions of discovery rate, transmission rate, and selective advantage are fundamental parameters of cultural evolution and have been well established in the literature at least since Boyd and Richerson's "Culture and the Evolutionary Process". In these traditions, cultural change is typically understood as being governed by (i) the rate at which novel variants are generated, (ii) the efficiency and fidelity with which they are transmitted, and (iii) the extent to which they confer differential success or attractiveness, particularly within social learning processes. Our proposed dimensions correspond directly to these foundational parameters: discovery difficulty constrains innovation, transmission difficulty constrains diffusion, and selective advantage constrains identification of successful teachers.

In the previous version of the manuscript, the connection between the different processes of cultural evolution and these constraining factors was insufficiently developed. In the revised manuscript, we now link the two much more clearly. We also agree with the reviewer that our earlier wording was imprecise, as these dimensions are more appropriately understood as

modulating the influence of machines on human culture rather than constituting strict necessary or sufficient conditions. Demonstrating sufficiency is difficult in real-world settings, as cultural dynamics are shaped by numerous interacting factors that no single model can fully capture. Similarly, establishing necessity for such complex phenomena in an all-encompassing manner is rarely feasible.

However, we believe that the reported results from our agent-based model, in conjunction with empirical evidence for the existence of machine-induced cultural shifts, are sufficient to suggest that discovery difficulty, transmission difficulty, and recognizable selective advantage constitute important moderators of machine-driven cultural change.

In response to this and related comments, we have redesigned the conceptual figure and strengthened our argument in the Introduction.

We correspondingly updated:

- **In the abstract:** *We identify three key dimensions that modulate machine influence on human problem-solving:*
- **In the introduction:** *We address this question by integrating insights from the field of cultural evolution [7]: culture changes through a process of variation, transmission, and selection [8, 9]. Accordingly, the space of culturally adopted strategies is constrained by human discovery capacity, transmission fidelity, and the ability to perceive utility [10, 11, 12]. We propose that machines' capacity to discover novel strategies can expand these boundaries, contingent on three critical factors. Appropriate discovery difficulty: …*
- ***Updated Figure 1B***

7/
The manuscript's structure blends introduction, design and results in a less conventional way; if this is intentional or aligned with the journal's style, discard this point

Following nature's general format, we place the method section at the end of the manuscript. Correspondingly, we have to introduce key elements of the design in the introduction in order for readers to be able to comprehend the results.

Minor comments

• When reporting regression results (of any type), please include p-values or at least a measure of model comparison or fit. Even if very large betas are almost certainly significant, it is preferable to document this explicitly.

In line with the recommendations of Bates et al. (2015) for linear mixed-effects models as implemented in lme4, we do not report p-values for fixed effects, as they are unreliable. Instead, we report bootstrapped 95% confidence intervals as per the preregistration. These provide the same inferential information (effects whose intervals exclude zero are statistically significant) while additionally quantifying estimation uncertainty. All reported effects have confidence intervals that exclude zero, and are therefore statistically reliable.
We have now clarified this analytical strategy explicitly in the section "Statistical procedure".

Bates, D., Mächler, M., Bolker, B., & Walker, S. (2015). Fitting linear mixed-effects models using lme4. *Journal of statistical software*, *67*, 1-48.

• The simulation-based power analysis is only briefly described. Please expand on how it was performed, especially if pilot data informed the parameter choices.

In general, power analyses for mixed-effects models are not straightforward, but sampling from simulated data provides a good solution to this problem (De Bruine & Barr, 2021). Rather than making assumptions about distributions independently of our novel task, we built an agent-based model similar to the one presented in the manuscript to create synthetic data for 100 independent runs of the full experimental design. Its parameters (rates of discovery and learning) were determined from pilot results.
The power analysis used this synthetic data by fitting our preregistered models on randomly drawn samples of different size (picking 10 or 15 replications from the data), and repeating this 1000 times each. We checked the parameters of both main registered models (1a and b), but based our final decision on model 1b because it has the lower power. We determined that a design with 15 populations per condition was enough to find the predicted effect for 1b in over 95% of samples (957 of 1000 runs with a t-value > 2), leading us to conclude that this would mean sufficient power.
In summary, the pilots did not directly inform the parameters of the power analysis, but the agent-based model we used to create synthetic data to run the power analysis on. We have added new details from this longer description into the manuscript.

DeBruine LM, Barr DJ. Understanding Mixed-Effects Models Through Data Simulation. *Advances in Methods and Practices in Psychological Science*. 2021;4(1). doi:10.1177/2515245920965119

• The procedure by which participants “recorded themselves” for future generations is not entirely clear. Did they write textual descriptions, use webcams, or something else? If written, did you examine whether surface features (e.g., spelling errors, verbosity) affected the likelihood that a strategy was preserved? How did the machine players produce their recorded demonstrations?

In this experiment, our primary objective was to ensure that differences in outcomes reflected the strategies discovered and solutions produced, rather than extraneous factors. Accordingly, we sought to control for potential confounds, such as variation in the speed with which solutions were entered.

To this end, all moves entered by human participants, as well as those selected by the machine agents, were digitally recorded and stored in a database. During replay, these moves were reenacted with fixed, standardized delays between successive actions. This procedure ensured that participants experienced temporally equivalent demonstrations, regardless of whether they learned from human or machine sources.

With respect to written strategies, participants documented their personal approaches before and after the Social Learning Phase. However, we deliberately chose not to display these reports to subsequent generations, as doing so could have introduced uncontrolled surface-level influences as the reviewer correctly pointed out. Instead, these materials were

collected exclusively for our preregistered analysis aimed at probing participants' understanding and reasoning (2b).

We have clarified these procedures more explicitly in the revised manuscript.

Specific changes:

- **Addition to methods:** *Replay delays between moves were equalized to eliminate timing differences between human and machine-generated demonstrations.*
- **Addition to methods:** *Participants also documented their personal strategy in written form, before and after the Social Learning Phase. These reports were collected solely for later analysis and were not presented to subsequent generations.*
- **Addition to the introduction:** *They then observed animations of the demonstrator's moves, replicated them, and subsequently solved the same networks before receiving final feedback, with this procedure repeated across four networks.*

• The paper would benefit from a brief limitations section (with or without a dedicated header) to acknowledge, for example, the constrained laboratory setting and the potential confound between cognitive bias and training time.

We agree with the reviewer that explicitly acknowledging limitations is an important contribution to the Discussion. While the previous version of the manuscript addressed several limitations implicitly—for example, in our discussion of the importance of efficiently verifying strategies and the effect of group size on retention—we recognize that these constraints were not always clearly foregrounded.

All experimental studies are subject to limitations related to ecological validity, participant reactivity, sampling bias, task specificity, and scalability. Rather than enumerating these well-established issues in a generic manner, we previously chose to focus on the constraints most directly relevant to the present work and to discuss them in greater depth. However, we agree that making some of these limitations more explicit strengthens the manuscript and improves its transparency.

Accordingly, we have revised the Discussion to more clearly articulate the constraints imposed by the controlled, time-compressed nature of an online experiment, and to consider how these conditions may interact with human cognitive biases. In particular, we now explicitly address the potential confound between training duration and bias expression.

Specific changes:

- **New to the discussion:** *As a controlled online experiment, our design necessarily reflects stylized conditions, with individual learning constrained to brief exploration periods that may differ from real-world settings. However, analyses of participants in generation 0 with greater learning opportunities provide no evidence that extended exploration budgets attenuate loss-related biases (see Supplementary Text).*

Reviewer #2 (Remarks to the Author):

The article investigates the conditions under which machine-induced cultural shifts occur using controlled behavioral experiments and computer simulations. The study makes an

important contribution by approaching the topic of cultural evolution with AI (simple reinforcement-learning agents). It is technically commendable that the authors conducted an experiment requiring about 40 participants per group (8 participants × 5 generations, in the human-population condition). I find this study meaningful.
While I have no concerns regarding the importance of the research question, there are several points the authors may wish to address to improve the manuscript regarding the analysis and interpretation that should be improved. Please find some comments and questions below. Because the document does not include line numbers, some of the following comments may be unclear regarding which parts are being discussed. I would appreciate it if the authors could include line numbers in the files when they have the opportunity to revise and resubmit the article.

We thank the reviewer for highlighting the value of our research and for appreciating its technical execution. We apologize for the missing line numbers in the original submission. We are grateful for the detailed feedback and have added line numbers in the revised manuscript. In the following, we respond to the reviewer's individual comments.

1. There is a gap between what the authors state in the abstract and introduction and what they test in their experiment. The authors suggest that three conditions are involved in machine-induced cultural shift based on cultural evolution theory: Appropriate Discovery Difficulty, Low Transmission Difficulty, and Recognizable Selective Advantage. In the abstract, they state, "Using a cultural transmission experiment and an agent-based simulation, we demonstrate that when these conditions are met, machine-discovered strategies can be transmitted, understood, and preserved by human populations, leading to enduring cultural shifts." However, these conditions are not manipulated in the experiment presented in the paper (the preregistration and the discussion mention pilot data, but from what I can see, it is not publicly available in the main paper). Of course, it makes sense to test the three conditions in a low-cost simulation and then test just one specific combination in a more expensive experiment. However, since a simulation and an experiment are not the same, what is demonstrated in the simulation has not been demonstrated in the experiment. Therefore, it may be inappropriate to state in the abstract and introduction that they have shown the importance of the three conditions using both the experiment "and" the simulation. Conversely, if they were to argue that the experiment and simulation are essentially the same, then the experiment would have been unnecessary, and the simulation alone would have sufficed.

We thank the reviewer for highlighting this potential misconception. We agree that, in the original version, the wording of the abstract and introduction could be interpreted as suggesting that all three proposed dimensions were jointly and directly manipulated in the behavioral experiment, which was not our intention.

Our empirical approach is deliberately multi-method. Due to the practical constraints of large-scale transmission experiments, we experimentally investigate the existence of a regime in which culture is shifted by machines. We then examine, through systematic numerical simulations, the conditions that moderate such cultural shifts.

In the revised abstract, we now clearly distinguish between the evidence gained from the experiment and that obtained from the agent-based simulations.

Revised Abstract: *We identify three key dimensions that modulate machine influence on human problem-solving: the discovered strategies must be non-trivial, learnable, and offer a clear advantage. Using a cultural transmission experiment, we demonstrate that when these conditions are met, machine-discovered strategies can be transmitted, understood, and preserved by human populations, leading to enduring cultural shifts. Conversely, using agent-based simulations, we show how machine influence is constrained in the absence of these conditions.*

2. In Figure 4, the dots represent individual populations (15 populations per condition), but the analysis reported in reference to Figure 4 seems to use each participant as the unit of analysis, which is misleading.

We thank the reviewer for raising this point. The design of our experiment implies substantial correlation in behavior within populations. Accordingly, while the unit of observation in our analyses is at the level of moves, trials, or participants, our primary inferential target is the population level.

Our statistical models were specified to represent the data at the lowest meaningful level. We therefore measure performance at the level of individual trials, congruency at the level of individual moves, and verbalized strategies at the level of participants. As the reviewer correctly notes below, however, failing to account for the nested structure of the data could lead to inflated Type I error rates. We therefore included random effects at all appropriate levels to capture these dependencies.

For visualization, it is not feasible to display the full nested structure of the data. This creates a trade-off. Plotting individual moves, trials, or participants is more faithful to the raw data, but visually inflates the number of seemingly independent observations and may convey an exaggerated impression of statistical power. Conversely, plotting population-level summaries omits some underlying variability, but ensures that each displayed point represents an independent observational unit.

A similar consideration applies to the computation of error bars (next comment). The preregistered models were designed for hypothesis testing, but not for visualizing uncertainty in generation-level summary metrics. We therefore adopted a conservative approach for Figure 4. Specifically, we first aggregated outcomes at the population level and then computed bootstrapped confidence intervals on these aggregated values. Because this procedure does not fully exploit individual-level information, it likely overestimates uncertainty, but it avoids introducing additional hierarchical modeling assumptions for the purpose of visualization.

Taken together, we appreciate the reviewer's comment, which prompted us to revisit and clarify these issues. We have added error bars to Figure 4 and now explicitly state that

population-level averages constitute our primary inferential target. This renders Figure 4 internally consistent. Dots represent aggregates over populations and are independent within generations. Diamonds indicate the average of these population means, and error bars reflect the uncertainty associated with this average.

We additionally present participant-level data on performance and alignment in the Supplementary Information. We consider them informative, as they reveal a bimodal distribution in which some participants adopt the optimal strategy, whereas others do not.

Specific Changes:

- Updated Figure 4: We added bootstrapped confidence intervals.
- Updated Figure S11: Rather than showing maximum performance within the population, which was not really informative, we are now displaying the individual performance of each participant.

3. In Figure 4, the authors might want to include standard errors or CIs around the means.

We thank the reviewer for drawing our attention to this issue. We agree that presenting error bars in Figure 4 is appropriate. We now report bootstrapped confidence intervals in Figure 4; see our response to the previous comment for further details.

Specific Changes:

- Updated Figure 4: We added bootstrapped confidence intervals.

4. In the preregistration, four models (1a, 1b, 2a, and 2b) were listed. The authors should clarify which of these models corresponds to the analysis they describe in the main text when they state, "Our preregistered linear mixed effects model on these latter generations supported that this difference was significant." Furthermore, if they used model 1a, they should explicitly state that it is a model that tests for an interaction. If they are reporting a model that does not include the interaction, they should also report the model that does include the interaction, as specified in their preregistration.

The main document now explicitly refers to the numbers of predictions/models from the preregistration that are being tested. We also explicitly mention the interaction effect in model 1a and 2b now. All reported models have been preregistered and implemented exactly as specified in the registration.

5. If individual participants are the unit of analysis, the model must explicitly account for the nesting of participants within trees and generations. Otherwise, there is a risk of a Type I error. Unfortunately, the preregistered models do not seem to include this hierarchical structure. I would recommend that the authors re-analyze their data with the appropriate nesting structures. If the model fails to converge due to its complexity, it suggests that the sample size was too small to begin with, and it is not a valid reason to ignore the hierarchy in the analysis

There is a lot to unpack here, which we structure into a two-part response:

1. Analyses that have participants as the unit of analysis (2b) structurally cannot fit a random intercept for participant level, because there is only one observation per participant. We do fit random intercepts for participants in the analyses that can fit them (1a-2a) because they are on the level of trials or even moves-within-trials.
   In these cases, the random effects in the models are indeed not explicitly nested. We are, however, accounting for the nesting in how we code participant ids (“implicit nesting”), which is statistically equivalent and recommended by Schielzeth & Nakagawa (2013). The way this works is that participants have a unique identifier (i.e. rather than participant 1 in population 1 and participant 1 in population 2, we have participant 1 in population 1 and no second participant 1), which lets us formally model nested effects as crossed effects and get the same results.
   So in our view, part of the answer is a misunderstanding about how the nested structures are implemented in the actual code. As an example, we fit the same model (1b, because it is the simplest showcase) with non-unique identifiers, but nested the effects, and get the exact same results:

```
Linear mixed model fit by REML ['lmerMod']
Formula: solution_total_score ~ condition + (1 | session_id) + (1 | branchID)
   Data: demo_lastgen

REML criterion at convergence: 14466.6

Scaled residuals:
    Min      1Q  Median      3Q     Max
-3.8143 -0.5611  0.1744  0.5973  2.3803

Random effects:
 Groups     Name        Variance Std.Dev.
 session_id (Intercept) 103598   321.9
 branchID   (Intercept)  33450   182.9
 Residual               144810   380.5
Number of obs: 960, groups:  session_id, 240; branchID, 30

Fixed effects:
               Estimate Std. Error t value
(Intercept)     1814.79      58.27   31.15
conditionwo_ai  -229.06      82.40   -2.78

Correlation of Fixed Effects:
            (Intr)
conditionw_ -0.707
```

```
Linear mixed model fit by REML ['lmerMod']
Formula: solution_total_score ~ condition + (1 | branchID/session_id)
   Data: demo_lastgen

REML criterion at convergence: 14466.6

Scaled residuals:
    Min      1Q  Median      3Q     Max
-3.8143 -0.5611  0.1744  0.5973  2.3803

Random effects:
 Groups              Name        Variance Std.Dev.
 session_id:branchID (Intercept) 103598   321.9
 branchID            (Intercept)  33450   182.9
 Residual                        144810   380.5
Number of obs: 960, groups:  session_id:branchID, 240; branchID, 30

Fixed effects:
               Estimate Std. Error t value
(Intercept)     1814.79      58.27   31.15
conditionwo_ai  -229.06      82.40   -2.78

Correlation of Fixed Effects:
            (Intr)
conditionw_ -0.707
```

2. The additional effect of generation in the hierarchy in models 1a and 2b is an interesting part not discussed in 1., and the reviewer’s comment encouraged us to revisit the random intercept structure here. The way we treated generation as continuous meant it was included in the hierarchy via a random slope, but not explicitly modeled as a grouping factor, which the reviewer’s comment seems to suggest. In our view, both approaches are legitimate, but put the emphasis on different roles for the variable: either on testing how participant performance develops through time in the different conditions (our original, preregistered analysis), or on fitting generations into the hierarchy more tightly, but letting them vary randomly (the new approach). As a robustness check, we reran models 1a and 2b with generations nested in populations instead of the fixed predictor variable for generation. In 1a, participants are in turn nested in generations (not possible in 2b, see above). We find that all models converge and the main effect of condition is robust also with this different random effects structure:
   Performance Model 1a: $\beta = -309.2$, $SE = 40.3$, $CI_{95\%} = [-418.0, -195.4]$
   Congruency Model 1a: $\beta = -0.89$, $SE = 0.14$, $CI_{95\%} = [-1.18, -0.63]$

Model 2b: $\beta = -3.97$, $SE = 0.53$, $CI_{95\%} = [-5.16, -3.07]$

We report these new robustness checks also in the supplementary material, section "Robustness Checks".

Schielzeth, H., & Nakagawa, S. (2013). Nested by design: model fitting and interpretation in a mixed model era. *Methods in Ecology and Evolution*, *4*(1), 14-24.

6. In the discussion, the authors claim, "Our pilot experiments illustrated the key challenge of achieving the right balance between discovery difficulty and transmissibility." If the authors wish to maintain this claim, they should report the pilot data publicly.

We understand that the reviewer interpreted this statement as reporting a result at the same epistemic level as our other findings, which was not our intention. Accordingly, we have revised the statement in the Discussion to clarify that this observation reflects an emergent insight from iterative pilot work rather than a definitive empirical conclusion. In its revised form, we believe the statement more accurately conveys both the empirical basis of this observation and its provisional character.

In addition, we now also explicitly refer to the qualitative description of our pilot studies provided in the Supplementary material within the main manuscript.

Specific Changes:

- Update in Discussion: <u>In pilot experiments, we found a key challenge in achieving</u> the right balance between discovery difficulty and transmissibility <u>(see Supplementary Text)</u>

Reviewer #3 (Remarks to the Author):

This paper examines the conditions under which machines can become drivers of (persistent) cultural change. It studies cultural evolution in populations of humans trying to solve a problem, vs populations of humans and machines; and tries to determine what conditions are necessary for machine-discovered ideas to spread *and* be fully incorporated by humans. It identifies three such conditions for solutions: they must have a selective advantage, they must be non-trivial, and they must be learnable. This is done with cultural transmission experiments, that are nicely combined with computer simulations in order to examine counterfactuals and establish these conditions are all necessary.

For the experiment, a specific task is designed such that humans have a bias that makes it hard to discover optimal solutions. Machines, on the other hand, can quickly find optimal solutions using standard learning algorithms. Around ~1.2k participants were split into 30 populations; half were Human-Only, and half were Human-Machine, including some "machine players" inserted in Generation 0. After practicing solutions, participants chose a demonstrator from five candidates shown with their average scores (without knowing who was human or machine), watched that replay, reproduced it, and finally solved new networks and recorded their own demonstration for the next generation. This design allows the authors to test whether an machine-discovered strategy would be learned by humans and preserved across generations even in the absence of machines.

The experiment is complemented with some agent-based simulations that mirror the lab setting. Here, each agent can pick a strategy (random, myopic, or optimal), and in each generation it either explored on its own or learned socially by picking a demonstrator from five candidates in the previous generation. The simulation allows control over discovery and transmission difficulty. The key result (Figure 5, p. 11) is that machines create a lasting boost only when the optimal strategy is hard for humans to discover but still easy to learn; if discovery is easy, learning is random, or transmission is too difficult, the gains provided by machine discoveries are small.

We thank the reviewer for their detailed and thoughtful engagement with our work. We are pleased that the reviewer appreciates the combination of transmission chain experiments and computer simulations. We are also grateful for the reviewer's insightful questions, which we address individually below.

Some minor comments:

- I think it would be interesting to tease out more when each condition is required. The fact that selective advantage is necessary but not sufficient is somehow puzzling. I suppose this has to do not only with ideas spreading, but the fact that they need to be incorporated by humans in the absence of machines. If that is the case, would machines teaching each other will again guarantee that selective advantage on its own is enough? I feel this could be spelled out more clearly in the discussion.

We thank the reviewer for this insightful point. In the present manuscript, we focus specifically on how machines may influence and reshape human culture. That said, we fully agree that there are many scenarios in which AI systems may maintain adaptive cultural artifacts and strategies without direct human involvement. Many AI systems are used primarily as tools (such as AlphaFold), and these systems may not influence human behavior, at least not in the sense that knowledge or strategies are directly transmitted between the AI system and the humans interacting with it.

Moreover, methods such as knowledge distillation demonstrate that information can be transmitted between AI systems in the form of cultural artifacts (in this case, text). Recently, these systems are increasingly shaped through experience, using methods from reinforcement learning. It is therefore foreseeable that scenarios will emerge in which AI systems produce some kinds of cultural artifacts describing behavioral strategies that are human-incomprehensible but are then used by other AI systems as the basis to adapt their behavior.

In conclusion, we greatly appreciated the questions raised by this argument. We have added a brief discussion of this issue to the Discussion section. In addition, the updated Figure 1B now more clearly features a domain beyond human understanding, which effectively illustrates this regime.

Specific Changes:

- **Addition to the Discussion:** *At the same time, pedagogical features in AI systems may not always be necessary or desirable, particularly when humans use these systems primarily as tools. As AI systems are increasingly trained on one another's outputs, they may transmit adaptive strategies in the form of cultural artifacts that are opaque to humans, potentially leading to a bifurcation of human and machine cultures [7].*
- ***Updated Figure 1B***

- Can the authors explain the appeal of the random strategy in the simulation? Would the same results arise without it?

In the experiment, we observed three broad classes of behavior: myopic, optimal, and other non-classifiable patterns. We therefore included a random strategy in the original model to reflect this heterogeneity. However, we agree with the reviewer that, in its initial formulation, the random strategy provided limited insight.

Following Reviewer 1's comments regarding the disentanglement of bias and exploration capacity, we substantially revised the agent model. In the updated version, agents can explore two distinct strategies (myopic and optimal). We also retained a null strategy to capture situations in which agents adopt neither the optimal nor the myopic strategy. The null strategy is functional similar to the random strategy in the original model, and serves as a fallback, should an agent fail to discover any strategy.

Although it would be possible to reformulate the model using only an optimal and a default (myopic) strategy without changing the results, we consider the current formulation more intuitive. It reflects the assumption that agents start without strategy, and then can choose to explore one of two alternative strategies. This requires a fallback option if they fail to discover either.

Specific Changes:

- Updated Model in Methods

- Can the authors comment on what they would expect with tasks that are more fair or where the advantage of machines is large but not huge? The task is particularly easy to solve for machines: not even Q-learning would be required for an optimal solution to be found.

At present, intelligent machines appear to outperform humans primarily under conditions of substantial informational or computational advantage. Large language models, for example, are trained on datasets that vastly exceed the amount of information any human is exposed to in a lifetime. Similarly, AlphaGo was trained on millions of games, far surpassing the number any human could ever play. With the exception of pretrained LLMs, machine learning systems generally seem to underperform compared to humans under more equitable learning conditions. The zero-shot capabilities of LLMs constitute a notable exception; however, these capabilities are themselves bootstrapped from human-generated culture.

In the present experiment, analogous to some versions of DeepMind's Go algorithms, the agent explores without leveraging prior human knowledge. Investigating the dynamics that

arise when such cultural bootstrapping is available is an intriguing direction for future work, but it lies beyond the scope of this paper. We therefore take the reviewer's comment as an opportunity to clarify these limitations in the Discussion and to highlight open questions for future research.

To illustrate how exploration advantage and machine bias modulate the expansion of obtainable strategies in hybrid populations, we expanded the parameter space explored in the agent-based model. The revised Figure 5 now depicts three levels of machine exploration advantage and two levels of machine bias. These results show that increasing machine advantage expands the discovery difficulty limit of adoptable strategies, whereas transmission difficulty remains a persistent constraint. This constraint can be shifted only by (a) more selective social learning strategies or (b) more persistent availability of machines (see Figure S13).

We agree that other algorithms could identify the optimal solutions on an individual network. However, to translate such solutions into a strategy that can be applied to novel networks, without having access to the network structure is less trivial. As the networks differed between training and testing, and because their structure was not observable to either human participants or the algorithm, discovering the optimal solution in a single instance was insufficient for inferring the appropriate strategy of initially accepting three large losses. Instead, the agent had to extract and generalize this insight across heterogeneous and previously unseen network realizations. Learning such a transferable, model-free policy under partial observability represents a nontrivial computational challenge, which we discuss in greater detail in the revised manuscript.

Specific changes to the manuscript:

- **Addition to the discussion**: *Our results apply particularly to scenarios in which human exploration is constrained, while machine exploration remains scalable. For instance, chemical recipes can be explored at high throughput and scale through automation [39]. In our experiment, the state space was small enough to permit brute-force exploration, and the primary role of Q-learning was to extract a generalizable, model-free strategy from this experience. In many real-world domains, however, solution spaces are intractable and exploration is resource-intensive. Future research should examine whether the extensive world knowledge embedded in large generative models might similarly facilitate previously inaccessible discovery processes when both humans and machines operate under comparable data constraints.*
- **Updated Figure 5:** Now depicting different levels of machine advantage
- **Updated Figure S13:** Now depicting how social learning strength and the presence of machines (non, temporarily, permanent) moderate adoption.

This is I think a great paper that combines a fantastic question with good methods. The topic is fascinating and will be of interest to computer scientists and social scientists alike. Overall, the paper convincingly shows that a machine-discovered, non-trivial but learnable strategies can spread and persist. It would be good to extend these to other less engineered tasks where the advantage of machines is less salient, but this may be part of the many extensions I am sure the work will inspire.

I recommend publication.

We thank the reviewer for highlighting the relevance of the question addressed and the appropriateness of our methods. We are likewise excited to see which follow-up research this work will inspire, particularly, as the reviewer notes, in domains where exploration advantage is less feasible.

Reviewer #3 (Remarks on code availability):

Code is complete and seems to be of high standard.

Reviewer #4 (Remarks to the Author):

This paper is a good, straightforward demonstration of what it sets out to do. The authors show that machines can discover solutions that are counterintuitive to humans, and that humans can adopt them. The experiment and the model seem solid, well executed, and clearly presented. I thought the fact that the agent-based model recovers the theoretical expectations presented in the introduction a nice touch. I don't find the fact that humans can recognise good solutions and use them particularly surprising, and I don't think it is my place to comment on whether the novelty of this result qualifies the paper for publication in Nature Communications. Below I summarise some concerns I have with the framing, and some suggestions for improving clarity.

We thank the reviewer for highlighting the rigor of our work, both in the experimental and modeling components. We are pleased that the reviewer appreciated our efforts to reproduce key results numerically, thereby strengthening the connection between our theoretical contributions and empirical findings. In the following, we respond individually to the remaining points.

One niggling question I have is how much the dynamic captured here is about machines specifically vs. humans being able to recognise and integrate new information in their strategies, and whether this difference even matters. If I read the results correctly (Fig. 3, S6), one human participant discovered the optimal strategy and passed it on to the next generation, but this took place at the end of the experiment (generations 3-4) so we don't know if this strategy would have persisted further. The expectation is that it would have. Would the human participants have discovered the optimal solution more often with enough time (6 trials seems low)? The novel point is then not that machines can transform human culture, but that machines can generate new solutions that humans can readily use.

We thank the reviewer for this thoughtful comment which also relates to an earlier comment by reviewer 1. We agree that the strategy discovered by the human participant in Fig. 3 and Fig. S6 would likely have spread further, had additional generations been observed. As the reviewer notes, once a high-performing strategy is discovered and successfully transmitted, its subsequent cultural trajectory no longer depends on whether it originated from a machine

or a human. This is central to our argument: cultural transmission operates on the properties of strategies, not on their origin.

Accordingly, our results support two core insights. First, machines, through greatly increased exploration capacity and reduced susceptibility to myopic or culturally entrenched biases, can discover strategies that humans may fail to uncover under realistic constraints, such as limited time and cognitive or cultural biases. Second, once such strategies are discovered, regardless of their origin, they can be transmitted, learned, and maintained within human populations through standard cultural evolutionary processes. The novel contribution of our work is to experimentally demonstrate this phenomenon, thereby opening up opportunities to study the conditions that mediate such cultural shifts.

The fact that machines can discover strategies that humans (under typical constraints) fail to identify, and that these strategies can subsequently be adopted by other humans, constitutes a form of cultural change. We have revised the Discussion to clarify this epistemological contribution.

We agree with the reviewer's implicit assumption that, given sufficient time, humans can in principle discover and master the optimal strategy. By analogy, laboratory studies of cumulative cultural evolution show that individuals may outperform multi-generational groups when total experience is equalized (Derex et al., 2024, https://doi.org/10.1073/pnas.2322886121). However, the central phenomenon of cumulative culture is that experience can be accumulated beyond the limits of a single lifetime, enabling strategies that no individual could realistically discover alone.

A similar asymmetry applies to machines. AI systems can explore at speeds and scales that exceed human capacities by orders of magnitude, reaching exploration budgets unattainable within a human lifetime. In the case of AlphaGo, for example, the system played on the order of a million self-play games. Although a human brain might, in principle, arrive at comparable strategies given sufficient exploration, this is infeasible in practice. Humans can accumulate such experience only culturally, at the cost of transmission noise and reconstruction across generations. Our experiments therefore deliberately constrain human exploration time to examine the impact of machines under these conditions.

In the revised manuscript, we clarify these design choices and underlying assumptions and emphasize the interplay between exploration time and exploration bias. In response to Comment 1 by Reviewer 1, we also report a new analysis showing that human exploration is biased toward myopic solutions, further hindering the discovery of the optimal strategy.

Specific Changes:

- **Clarification in the Discussion:** This does not mean that the optimal strategy was impossible to discover for human participants at all, <u>but limitations in time and cognitive biases</u> made it exceedingly rare. Yet, the machine-discovered strategies were still learnable for human players, who did not merely replicate them but also applied them to new variations of the task. Further signifying the integration of the counterintuitive problem-solving strategy into their cognitive framework, many human players explicitly articulated the strategy in writing. The machine-discovery's selective advantage translated into human players overwhelmingly favoring machine over

human ancestors, producing persistent cultural lineages where every human player in the last generation was ultimately a descendant of a machine player in the first generation. Consequently, the optimal strategy was adopted by the majority of populations with machine ancestors, whereas human populations predominantly employed the myopic strategy, indicating the capacity of machines to act as drivers of cultural change.

- **Specification of assumption in the introduction**: *Our experimental setup reflects the cognitive heterogeneity and asymmetric exploration capacities of human-machine societies, thereby extending prior work which typically considers one agent type.*
- **Clarification in the Discussion:** *In our experiment, exploration at multiple orders of magnitude greater capacity allowed machine players to discover a superior strategy that offset human cognitive biases toward myopic and suboptimal solutions and, as such, was effectively beyond human reach within the constraints exposed by the experiment. This does not mean that the optimal strategy was impossible to discover for human participants at all, but limitations in time and cognitive biases [1] made it exceedingly rare.*
- **New to the Discussion**: *Human culture supports the cumulative accumulation of knowledge and skills across generations, enabling individuals to benefit from collective experience that far exceeds what any single person could acquire in isolation [10, 9]. Laboratory studies explicitly compressing generational timescales into hours or minutes show that social transmission can overcome time constraints and the costs of individual exploration [37, 38]. Building on this literature, the present work suggests that machines may function as novel sources of cumulative cultural input. By learning from machines, humans may adopt and transmit hard-to-conceive optimal strategies, thereby expanding their cognitive repertoire without incurring the cognitive costs and time typically associated with discovering such strategies.*
- **Completely new analysis in SI**: Supplementary Text, Estimating Human Exploration Bias

Another point that I think should be discussed directly in the paper is the role that payoff-bias (i.e. the fact that humans have access to score and can use score to dictate their social learning) plays for these results. I am assuming that participants chose the highest-scoring model most of the time (it would be nice to see some numbers on this), which again means humans have built-in mechanisms to take advantage of good information and good solutions. The phrasing in the results section is potentially misleading. The section titled "Humans Preserve the Machine Discovery" seems to imply there is something special about the machine solution that ensures it is preserved, and I suspect the something special is better score. I suggest the authors mention this directly in the text (or better yet, run additional analyses looking at the relationship between score and transmission). Similarly, the next section "Humans are Behaviourally Congruent with the Machines" seems to suggest the human participant intend to copy the machines – would you find the same congruence with the loss strategy discovered by the one human participant in the human-only condition?

We thank the reviewer for raising the insightful question regarding a payoff-based selection of demonstrators. Previously, we had not analyzed this explicitly, assuming, as the reviewer suggested, that a high fraction of participants would choose the highest-scoring model most of the time. Our new analysis confirms this intuition: in 86% of cases, participants chose the

highest-scoring demonstrator. We have added this important information to the Results section in the revised manuscript.

Regarding the title "Humans Preserve the Machine Discovery," we appreciate the reviewer's concern about potential misunderstandings. We do not intend for the title to imply an inherent feature of the strategies that ensure preservation; in fact, our experiment was designed to investigate the inverse. The optimal strategy discovered by the machine conflicts with known human biases, making it potentially more difficult to preserve. To avoid similar misunderstandings, we have revised the title to: "Humans Preserve the Optimal Strategy Discovered by Machines."

Similarly, we acknowledge the concern regarding the title "Humans are Behaviourally Congruent with the Machines." Our use of "congruent" is not meant to imply intent, but rather to describe the empirical fact that human behavior in the hybrid conditions aligns more closely with counterfactual machine behavior (which we computed for each human action). We agree that participants who discovered the optimal strategy (and those in subsequent generations) demonstrate similar congruency with machine behavior. Indeed, Figure S9 in the Supplementary Material illustrates this effect. We have now explicitly incorporated this result into the main manuscript.

Specific Changes:

- **Addition to the results:** Preservation of the optimal strategy was supported by a strong bias toward selecting the demonstrator with the highest payoff. Overall, 86.7% (CI_95%=[84.5, 88.7]) of all participants chose the highest-scoring demonstrator.
- **Updated subsection title: "**Humans Preserve the Optimal Strategy Discovered by Machines"
- **Addition to the results:** *Notably, the individual who independently discovered the optimal strategy, as well as subsequent participants, likewise exhibited elevated congruency with machine behavior (see Supplementary Figure S9).*

Minor comments below:
• On page 7 please mention how many training trials generation 0 humans get, and mention how many individual learning trails generations 1-4 get and whether is it before/after social learning

We thank the reviewer for highlighting these gaps in our description of the experimental procedure. We have now updated the description to include information on the number of trials and the sequence of the experiment.

Specific Changes:

- Updated Introduction: After a series of <u>six</u> individual attempts at solving networks, participants in Generation 0 solved four networks during the "Demonstration Phase," knowing that their moves would be recorded for future generations to learn from. <u>After two individual trials to familiarize themselves with the tasks,</u> participants from Generations 1–4 could choose a "demonstrator" from the previous generation to learn from, based on the demonstrators' average performance. They did not know the demonstrators' identity as a human or machine, but were informed that machine

players were a possibility. They then observed animations of the demonstrator's moves, replicated them, and subsequently solved the same networks before receiving final feedback, with this procedure repeated across four networks. Only after this Social Learning Phase did participants from Generations 1–4 demonstrate their moves for the next generation.

• Page 9 – "Panel B in Figure 4 shows the proportion of human moves congruent with machine-generated moves, summarized by generation and population." – which machine generated moves?

We thank the reviewer for highlighting the ambiguity in the previous version of the manuscript. In the earlier submission, we computed alignment with respect to the first machine only. Given that, in the experiment, solutions were generated by three distinct machines, this approach was somewhat inconsistent. Empirically, the three machines produce convergent outputs in approximately 90% of all moves. In the revised manuscript, all plots are therefore computed based on alignment with the majority vote of the three machines.

Specific changes:

- **Updated figure 4**
- **Updated figure caption:** Behavioural Congruency (Panel B) indicates the proportion of human player moves that are congruent with the majority behavior of the three trained machines, contingent on making the same previous moves.
- **Updated the Methods:** We updated the paragraph on Behavioural Congruency to reflect this change.
- **Updated Results Tables:** We updated the result tables on Behavioural Congruency to reflect this change.

• Fig. 4c – why do human-only participants in generation 0 mention a loss strategy and how did this translate into the participants' score?

This is a good observation by the reviewer: In total, 7 out of 120 participants in generation 0 in human populations got coded as "mentioned the loss strategy" in the data. That this number is not 0 may be surprising, but it pales in comparison to the 62 participants who mention the strategy in the first generation of the human-machine condition (which directly learned from AI – but only from its presented solutions, putting a strategy into words was up to them).

Writing down and then coding written strategies is an inherently interpretative process, and it is unclear whether these participants just voiced suspicions, actually figured out the strategy, or whether this is just noise in the data or in the coding process. A qualitative inspection of the strategies suggests that they reasonably reflect the loss strategy, at least as defined by our coding manual. Of course, the calibration of the manual itself is arbitrary and could have ended up with less or more "positive" codings depending on the choices made before rating the data. The important part is that comparatively, participants differ from generation 1 onwards. Most participants in generation 0 also remained vague, e.g. "you have to reduce some points to gain some", suggesting that at least the exact implementation of taking three losses might have been hidden from them.

Most importantly, the hunch that they might have had did not translate into better performance. The 9 players range from 1000 to 1600 points total, whereas we expected more than 2000 points for a player implementing the loss strategy (see “Humans Preserve the Machine Discovery”). Crucially, also their demonstration trials do not look much better, with only two select trials just scratching the 2000 point mark, but many failed attempts demonstrating 1500 points or less. So even by the end of the experiment, they were not able to demonstrate that they learned a superior strategy.
In summary, it is hard to pinpoint down exactly whether these selected participants had a true understanding of the strategy or whether the result was a mere artifact of the coding process, highlighting its limitations; but they evidently did not have the impact on subsequent participants (or even their own performance) that they should have had, had they truly mastered the loss strategy.

Specific changes:

- Added this discussion to the qualitative description of strategies

• Figure 5a – I struggled to parse this description, consider adding more detail

We thank the reviewer for highlighting the difficulty in comprehending the original Figure 5a. In light of this and related comments, we realized that Figure 5 was generally hard to read and interpret. Accordingly, we decided to completely redesign Figure 5. We believe that the heatmaps presented in the revised manuscript are much easier to interpret, and that the corresponding description now reads considerably more smoothly.

Specific Changes:

- Updated Figure 5; updated figure caption

• Page 12 – in the discussion on population size and complex solutions, I would argue that the kind of phenomenon described here might benefit smaller populations more. Larger populations might have a higher chance of discovering the optimal solution, which would make the machine intervention redundant. It all depends on how novel/outside the human range the machine-discovered solutions can be.

We thank the reviewer for these very insightful observations. We fully agree that the interaction with population size is more complex than suggested in the previous version of the manuscript. We gave this point considerable thought and concluded that the relationship between population size and machine-induced cultural shifts depends on the particular limiting factor at play. Because stochastic loss is more likely in smaller populations, machine-induced cultural shifts should occur more frequently in large populations when transmission is limiting, and more frequently in small populations when discovery is limiting.

We updated the discussion to highlight this more complex relationship.

Specific changes:

- **Updated Discussion:** [...] Prior work in cultural evolution indicates that large effective population sizes can buffer against the loss of complex discoveries

> (Henrich, 2004; Derex et al., 2013), suggesting that machine-induced cultural shifts may be amplified when large groups interact with machine-generated strategies and transmission is the main constraint. [...] By contrast, in smaller populations, machines' advantages in discovery can be particularly pronounced, given the limited collective capacity of such human groups. Likewise, emerging evidence suggests that machines' ability to rapidly identify adaptive solutions within a population can enhance collective efficacy (Ueshima et al., 2024).

• Sentence "Our results apply particularly to scenarios in which human exploration is constrained by time rather than computational cost, machine exploration remains scalable" on page 14 might need editing

We agree that the previous sentence was confusing. In response to this and other reviewers' comments, we substantially revised the corresponding paragraph. The specific sentence was shortened to:

> *Our results apply particularly to scenarios in which human exploration is constrained, while machine exploration remains scalable.*

• The choices of parameter values for the agent-based model require justification beyond the fact that they nicely produced the curve in Fig. 2b – why the chosen d and t values, why was the discovery rate for machine agents set to 1000 higher than human agents, what does superior mean in "Upon discovering a new strategy, the agent updated its preferred strategy if the new strategy was superior to the previous one."

We revised the agent-based model (ABM), Figure 1B, and provided additional justification and parameter scans in the revised manuscript. In the original submission, we aligned the illustrative figure with the outcomes of the ABM for conceptual clarity. However, these components serve distinct purposes: the conceptual figure conveys a theoretical argument, whereas the ABM is designed to test and extend the conditions under which the experimentally observed results hold. We have therefore revised Figure 1B into a more stylized and explicitly conceptual form, disentangling the presentation of numerical results from the underlying theoretical framework.

We appreciate the concern regarding the assumption of a 1000× higher discovery rate for machine agents. In the model, the discovery rate scales the number of independent exploratory trials an agent can perform per generation; a 1000× advantage thus corresponds to evaluating three orders of magnitude more candidate strategies within the same generational window. Drawing on examples such as AlphaGo, which played millions of games within days, and on observed differences in exploration speed in our experiments, there is strong empirical motivation to assume that machines can, in some domains, explore multiple orders of magnitude faster than humans. The 1000× factor was intended as an illustrative benchmark emphasizing that, despite this advantage, machines may still face constraints. Given the centrality of this parameter, we now present three exploration regimes (1×, 100×, and 10,000×) in the updated Figure 5.

Regarding the transmission parameter t, the original range (0.7–1.0) was partly motivated by space constraints in Figure 1B and by an emphasis on high transmission loss. To incorporate regimes of lower transmission loss, we now adopt a broader range from 0.5 to 1.0.

For the discovery difficulty parameter d, we depict a wide logarithmic range from 1 to 10^6. Differences of several orders of magnitude in exploration scale are plausible in many domains, motivating a corresponding range for discovery difficulty.

We further clarify that agents prioritize the optimal strategy over the myopic one. When an agent discovers both strategies, it subsequently demonstrates the optimal strategy to the next generation. Although we considered a payoff-dependent transmission rule, evolutionary selection in our model operates primarily during the social learning phase, which is modulated by a temperature parameter controlling selection strength among teachers. For simplicity, we therefore assume that agents select the optimal strategy for demonstration. This choice is now described and motivated more clearly in the revised manuscript.

Finally, we conducted extensive parameter scans and robustness analyses. In the Supplementary Information, we examine the ability of homogeneous populations with varying exploration capacities and biases to discover and adopt the optimal strategy, analyze the effects of selection strength, reward noise and presence of machine agents.

We also report robustness checks across alternative population sizes and numbers of machine agents, showing that the qualitative pattern is retained.

Specific Changes:

- **New Methods include:** *Provided that evolutionary selection operates through teacher choice, we adopt this simple prioritization of demonstrated behavior, assuming that agents aim to maximize individual performance during demonstration.*
- **Updated Figure 1B**: The figure is now stylized and independent from the ABM
- **Updated Model description (Main Manuscript and Supplementary Text):** In the model description we describe and motivate all relevant parameter choices that we do not manipulate.
- **Updated Result section:** We have completely rewritten the ABM results section to build more systematically on a series of iterative numerical experiments, thereby more clearly motivating the manipulated parameters and the specific assumptions under which our results hold.
- ***New Supplementary Figures S12, S13, S14 and S15***

Reviewer #5 (Remarks to the Author):

I co-reviewed this manuscript with one of the reviewers who provided the listed reports. This is part of the Nature Communications initiative to facilitate training in peer review and to provide appropriate recognition for Early Career Researchers who co-review manuscripts.

Reviewer #5 (Remarks on code availability):

I co-reviewed this manuscript with one of the reviewers who provided the listed reports. This is part of the Nature Communications initiative to facilitate training in peer review and to provide appropriate recognition for Early Career Researchers who co-review manuscripts
